# Reverse engineering molecules from fingerprints through deterministic enumeration and generative models

**DOI:** 10.1186/s13321-025-01074-5

**Published:** 2025-10-15

**Authors:** Philippe Meyer, Thomas Duigou, Guillaume Gricourt, Jean-Loup Faulon

**Affiliations:** 1 Université Paris-Saclay, INRAE, AgroParisTech, Micalis Institute, 78350 Jouy-en-Josas, France; 2 The University of Manchester, Manchester Institute of Biotechnology, Manchester, M1 7DN UK

**Keywords:** Reverse engineering, Molecular fingerprint, Deterministic enumeration, Generative model, Drug design

## Abstract

Reverse engineering in molecular design aims to identify optimal structures based on activities, or properties, computed through molecular descriptors like fingerprints. This task is known to be particularly difficult for the widely used Extended-Connectivity Fingerprints (ECFPs), due to significant loss of structural information during vectorization. While recent artificial intelligence-based works have raised awareness about the privacy risks associated with ECFP-based data sharing, we contribute a more conclusive demonstration by introducing a deterministic algorithm that reconstructs molecular structures from ECFPs. Using MetaNetX and eMolecules as databases of natural compounds and commercially available chemicals, the deterministic algorithm benchmarks a Transformer-based generative model trained to predict SMILES from ECFPs. The generative model achieves a top-ranked retrieval accuracy of 95.64% but struggles with exhaustive enumeration. Additionally, applying the deterministic method to a drug dataset reveals its potential for de novo drug design, as many of the reverse-engineered structures are found to be patented or supported by bioassay data.

**Graphical Abstract:**

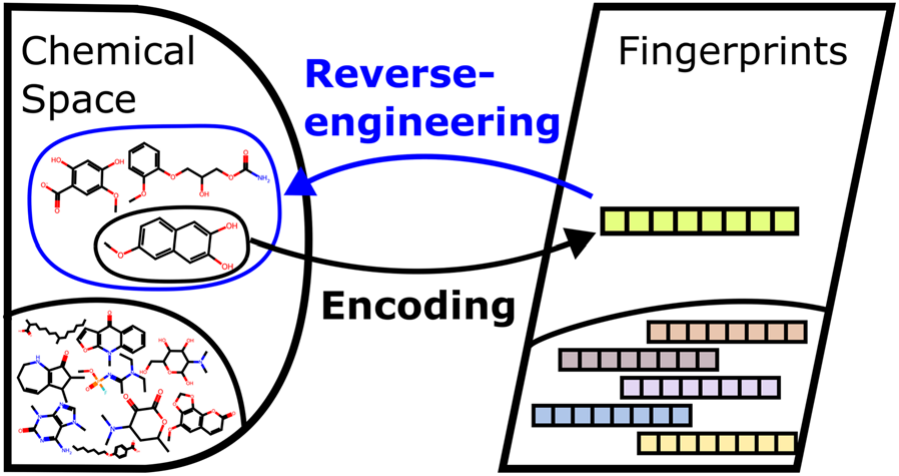

**Supplementary Information:**

The online version contains supplementary material available at 10.1186/s13321-025-01074-5.

## Scientific contribution

We present a deterministic algorithm that reconstructs molecular structures from ECFP vectors, demonstrating that these fingerprints are invertible. In parallel, we benchmark a Transformer-based generative model trained to predict SMILES from ECFPs, showing high accuracy but limitations in chemical space coverage. This dual approach advances reverse engineering in molecular design, offering new tools for de novo drug discovery.

**Supplementary Information:**

The online version contains supplementary material available at 10.1186/s13321-025-01074-5.

## Introduction

The design of molecules with specific desired properties has a longstanding history [1, 2]. This problem, known as reverse engineering, inverse Quantitative Structure–Activity/Property Relationship (QSAR/QSPR) problem or inverse design, begins with an intended set of functionalities as input and searches for ideal corresponding molecular structures as output.

High-throughput virtual screening emerged as an empirical alternative to directly solving the inverse QSAR/QSPR problem. Initially developed for drug discovery [3], this method aims to rapidly evaluate large libraries of chemical compounds to identify promising candidates. In contrast, optimization strategies offer more efficient exploration of chemical space and can generate novel compounds. For example, the imitation of the natural selection mechanism has led to the development of genetic algorithms that evolve a population of candidate molecules over successive generations through processes such as selection, crossover, and mutation [4, 5]. However, the stochastic aspect of the method may not find all solutions converging on suboptimal solutions.

Exhaustive enumeration overcomes these limitations by systematically generating all possible molecules that meet specific constraints, but becomes computationally expensive with increasing molecular complexity. An outcome of the graph-theoretic approaches, introduced in the nineteenth century [6] and developed up to the present day [7–9], is the development of chemical structure generators producing molecular graphs that obey chemical constraints [10–12]. The Generated DataBases (GDB) [13, 14] are notable examples of this strategy, systematically enumerating billions of chemically valid small molecules using graph-theoretic principles and chemical stability filters.

The signature molecular descriptor represents compounds by encoding local atomic environments and has been used to perform exhaustive enumeration [15]. More precisely, this molecular representation is defined as the collection of local subgraphs surrounding each atom, extending up to a specified radius $r$, which are typically expressed using SMARTS strings [16]. In this context, inverse QSAR problems are transcribed as rearranging atomic signatures [17], which is carried out by solving linear Diophantine systems [18–20]. Deterministic approaches are relatively rare, with most software implementations relying on stochastic methods for structure optimization [21].

Although significant progress has been made to reverse engineering, the chemical space remains excessively vast and difficult to explore [22]. For this reason and the fact that artificial intelligence continuously enhances its performance through experience, deep learning models have been employed and integrated with various approaches to the inverse QSAR problem, such as high-throughput virtual screening [23], genetic algorithms [24], chemical language models [25] and conditional generative models [26]. Generative techniques have also been applied in various ways to de novo drug and molecular design, providing alternative strategies for exploring chemical space [27–31].

The family of molecular fingerprints is one of the most commonly used molecular representations to express physicochemical or structural properties of molecules in machine-readable format [32]. This vectorization enables fast and accurate comparisons between molecules, helping to identify compounds with similar properties or biological activities, which is critical for cheminformatics and bioinformatics tasks such as drug discovery [33], QSPR modeling [34] and retrosynthesis [35]. There are several types of molecular fingerprints, each capturing different structural features, and they are often combined to enrich molecular representations [36, 37]. Among them, the most widely used is the Extended-Connectivity Fingerprint (ECFP) [32, 38] that iteratively captures and hashes local environments around atoms up to a specified radius $r$, to generate a fixed-length vector. The ECFP vectorization can be viewed as a hashed and folded analogue of the molecular signature descriptor.

Reverse-engineering molecular fingerprints is known to be challenging and commonly considered non-invertible [39], as the vectorization process inherently results in a lossy compression of information. Prior to the advent of modern generative models, this limitation was leveraged as a safeguard to prevent the disclosure of sensitive molecular information during data exchange [40]. However, recent advances in deep learning have revisited the reverse-engineering fingerprint problem, prompting explicit warnings about the risks of sharing such data between entities [41]. For example, Maragakis et al. [42] designed a neural machine translation architecture which is inspired by vector-to-sequence models and composed of a recurrent neural network (RNN) encoder combined with a long short-term memory network (LSTM) decoder to predict SMILES from ECFP. Similarly, Le et al. [43] employed a feedforward neural network to map the ECFP input into a continuous, data-driven molecular descriptor intermediate space, followed by a decoder RNN to predict the corresponding SMILES. Ucak et al. [44] noticed that the attention mechanism [45] is suitable for the use of Transformer models to decode fingerprints into lossless molecular representations. We also note that genetic algorithms together with machine learning have also been used to decode fingerprints [46].

In this work, we compare a deterministic enumeration algorithm with a deep learning-based Transformer model for generating molecular structures from ECFPs. Our analysis focuses specifically on ECFPs with a radius of 2 and a size of 2048 bits. The evaluation is conducted using two distinct datasets: the MetaNetX database, which comprises natural compounds derived from genome-scale metabolic networks and biochemical pathways, and the eMolecules database, which is composed of commercially available chemical compounds.

The exhaustive deterministic enumeration algorithm is composed of two main steps. Using an alphabet constructed from a molecular database that links atomic signatures to their Morgan bits, the signature-enumeration algorithm computes molecular signatures from ECFPs by solving linear Diophantine systems. The molecule-enumeration algorithm then reconstructs molecules from molecular signatures by extracting key atomic and bonding constraints from atomic signatures. Using test compounds from the MetaNetX and eMolecules databases, we show that this deterministic approach enables complete molecular reconstruction from ECFPs, given the appropriate alphabet and threshold settings.

The Transformer-based generative model we used to compare with the deterministic enumeration, is designed to predict SMILES strings from ECFP vectors and is similar to approaches such as the MolForge model published in Ucak et al*.* but it differs in key aspects, including the handling of counted ECFPs and its focus on different regions of chemical space. Transformers excel in sequence modeling by leveraging a self-attention mechanism to capture intricate dependencies and efficiently handle long-range relationships in data. Unlike sequential models, they process input in parallel, enabling faster and more comprehensive analysis.

In the following results, we first examine the distribution of ECFPs and molecular signatures across chemical databases, assessing the representativity of the alphabet connecting atomic signatures to Morgan bits within the chemical space. Next, we illustrate and compare the outcomes of the deterministic enumeration and the generative methods, using both specific examples and large datasets. Finally, we demonstrate the application of the deterministic method to the DrugBank dataset, highlighting its potential utility in drug design. For this purpose, we construct a unified alphabet by merging the MetaNetX and eMolecules molecular fragments, further enriched with ChEMBL to improve drug-like properties.

## Results

### ECFPs and molecular signatures distributions in large chemical databases

**Fig. 1:**
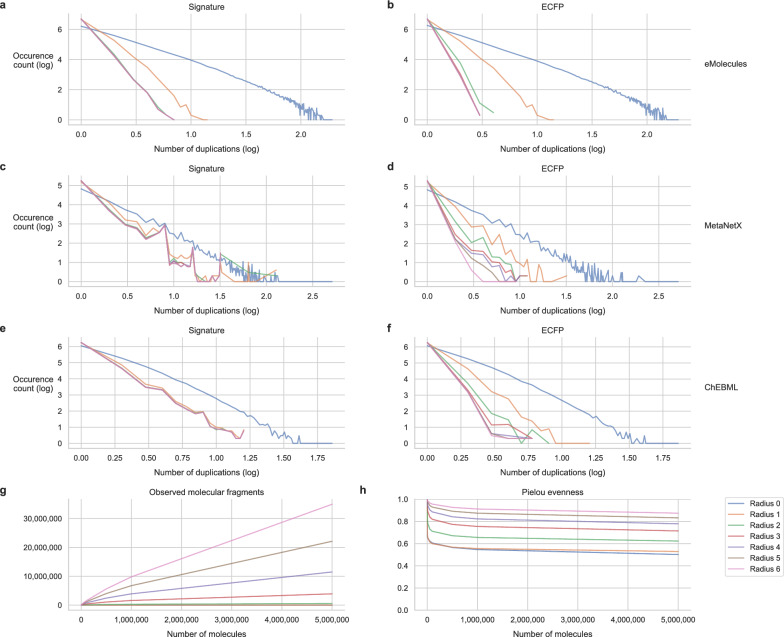
Number of molecules sharing the same molecular signatures or ECFPs and the evolution of the alphabet size. **a**–**f** Number of molecules sharing the same molecular signatures or ECFPs (x-axis) and the evolution of number of duplications (y-axis). The x-axis represents the number of duplications of a descriptor, while the y-axis indicates the frequency of these duplications. Both axes are presented on a log–log scale, in the sense that a point (x, y) corresponds to ${10}^{y}$ occurrences of a descriptor being duplicated ${10}^{x}$ times. **a**, **b** Duplication analysis for molecular signatures and ECFPs in the eMolecules database. **c**, **d**, **e**, **f** Similar duplication patterns for molecular signatures and ECFPs in the MetaNetX and ChEMBL datasets. **g** Evolution of the alphabet size (y-axis), analysed by incrementally sampling from 5 to 5 million molecules (x-axis) from the eMolecules database in 11 steps, with ten repetitions to track the growth of newly added signature fragments. **h** Pielou’s evenness metric (y-axis) used to evaluate the diversity of signature fragments across the sampling range (x-axis)

To analyse the distribution of ECFP and molecular signature representations, we computed these descriptors for all molecules in the MetaNetX [47] and eMolecules [48] databases, which are used in the two sections “Generation vs. enumeration of molecules from ECFP”, as well as for the ChEMBL database, used in the section “Application of the deterministic enumeration: drug design” (Methods “Datasets and sanitization”). As illustrated in Fig. 1, both descriptors exhibit similar patterns. Most of the generated representations are unique and varying the radius $r$ reveals that as the radius increases, both representations become more discriminative (Fig. 1a-f). Conversely, varying the length of the ECFP representation has little to no impact on this trend (Fig. S1). We also note that the distribution of molecules sharing the same molecular signature or ECFP descriptors remains nearly unchanged as the radius increases from 3 to 6 (Fig. 1a–f).

The MetaNetX database primarily consists of natural compounds, the eMolecules database contains commercial chemicals, and the ChEMBL database focuses on bioactive, drug-like molecules. An analysis of the frequency of atomic signature repetitions within molecular signatures reveals that the MetaNetX molecules exhibit a higher prevalence of identical local environments (Fig. S3a). Additionally, a TMAP visualization [49] comparing the three datasets highlights both significant distinctions and areas of overlap. Molecules from ChEMBL predominantly define the overall structure of the chemical space, forming a dense and interconnected backbone while eMolecules and MetaNetX contribute additional peripheral branches and localized clusters, reflecting their more specialized and partially distinct chemical subspaces (Fig. S2).

The alphabet that links atomic signatures to Morgan bits is a crucial component of the enumeration algorithm that associates molecular signatures from ECFPs (Methods “Enumerating molecular signatures from ECFPs”). To analyse its representativity in the chemical space, we computed the evolution of the alphabet size as the number of molecules from the eMolecules database used in its calculation increases, as shown in Fig. 1. For small radii, the growth of the alphabet slows after processing 1 million molecules (Fig. 1g), whereas for radii of 4 and above, it continues to expand dramatically. Using Pielou’s evenness index [50] (Fig. 1h), we found that as the radius increases, the distribution of atomic signature-Morgan bit tuples becomes more balanced, reaching a plateau quickly for each radius. At radius 2, the focus of this work, the first 100,000 molecules introduce an average of 9.64 new alphabet elements each. However, as the dataset expands to 500,000–5 million molecules, this rate declines to just 0.28 per molecule. Similarly, the proportion of molecules introducing new elements into the alphabet decreases to 2% after processing the dataset (Fig. S3c, d). These results show the high degree of chemical space representativeness achieved by the alphabet in radius 2 when a sufficiently large dataset is processed.

The eMolecules database is 24 times larger than the MetaNetX database, and its alphabet comprises 570,421 elements, which is only 2.4 times larger than the MetaNetX alphabet of 227,717 elements. The ChEMBL database, although 9.5 times larger than MetaNetX, yields an alphabet of 712,244 fragments, which is 3.1 times larger than the MetaNetX alphabet. The Venn diagram of alphabet overlaps (Fig. S3) reveals that each dataset contains a large set of unique fragments, with 404,497 fragments specific to ChEMBL, 292,074 to eMolecules, and 106,471 to MetaNetX. Pairwise overlaps vary from 6,638 fragments shared only between MetaNetX and eMolecules, to 193,139 between eMolecules and ChEMBL, while 78,570 fragments are common to all three datasets. These results highlight both the complementarity and the redundancy of the fragment spaces, which span several orders of magnitude across the different datasets.

Rogers et al*.* [32] reported that the number of 2-radius ECFP features computed for 50,000 randomly selected compounds was approximately 50,000 for the Asinex and Maybridge databases, and 100,000 for the World Drug Index. In our case, for 50,000 randomly selected molecules, we obtain approximately 109,000, 73,000, and 128,000 (Morgan bit, molecular fragment) tuples for the MetaNetX, eMolecules, and ChEMBL databases, respectively. This indicates that our representation captures a higher level of chemical diversity and encodes richer structural information than ECFP features alone.

### Generation vs. enumeration of molecules from ECFP through examples

In this section, we use the deterministic enumeration and the generative model to reverse-engineer ECFPs on specific examples. We have first chosen from the MetaNetX database the 2-Methyl-1,8-octanediol molecule represented by the SMILES string “C[C@H](CO)CCCCCCO”.

**Fig. 2:**
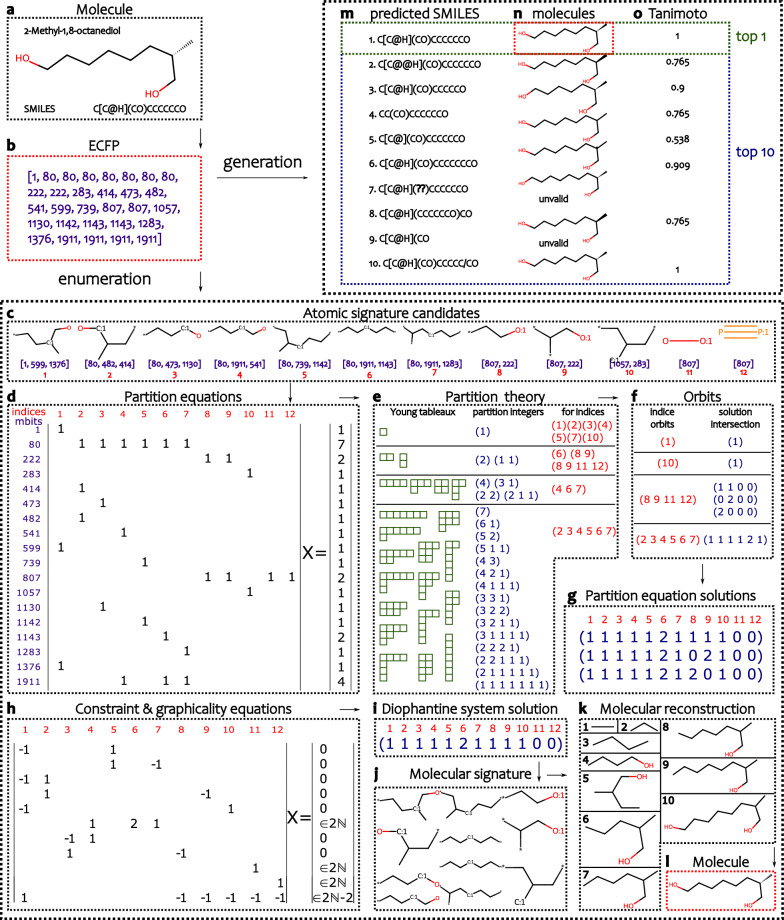
Example of the enumeration algorithm and the generative model on a specific ECFP. The step-by-step illustration of the enumeration algorithm applied to the ECFP representation of 2-Methyl-1,8-octanediol reads sequentially from **a** to **l**. **a** The 2-Methyl-1,8-octanediol molecule. **b** The input ECFP representation, shown as a counted list of its non-zero components. **c** From an alphabet created using the MetaNetX database, 12 atomic signatures are selected as potential candidates. **d** Partition equations are formulated based on the atomic signature candidates. **e** Young tableaux and integer partitions are used to find solutions per equation. **f** Four index orbits are identified among the indices involved in partition equations. **g** These orbits yield three solutions to the partition equations. **h** Constraint and graphicality equations are defined based on the atomic signature candidates. **i** Among the three solutions to the partition equations only one solution satisfies to the constraint and graphicality equations. **j** The valid solution produces a final molecular signature consisting of 11 atomic signatures. **k** The molecule-enumeration algorithm reconstructs a molecular structure by starting from a null graph of atoms and adding constraint links derived from the molecular signature. **l** The stereoisomer-enumeration produces two distinct molecular structures, one of which has an ECFP matching the input ECFP. Atomic signatures are depicted as rooted graphs, with the root node uniquely identified using the “:1” notation. The results of the generative model applied to the ECFP representation of 2-Methyl-1,8-octanediol are given in (**m**–**o**). **m** Top 10 predicted SMILES generated by the model. **n** Valid molecules among the predictions, with 8 out of 10 being valid. **o** Tanimoto coefficients of the predicted molecules relative to the input ECFP, ranging from 1 to 0.538. The first predicted molecule (top 1) matches the expected query molecule, while the tenth molecule is identified as a duplicate of the first by RDKit

We now outline the signature-enumeration algorithm on this ECFP with the alphabet computed from the MetaNetX database and provide a detailed illustration in Fig. 2a–l. The $2048$-bit ECFP4 vectorization of the molecule (Fig. 2a) has 18 non-zero components, with a vector sum equals to 30 (Fig. 2b). The signature-enumeration algorithm proceeds in several steps (Methods “Enumerating molecular signatures from ECFPs”). First, from the precomputed alphabet of atomic signatures linked to Morgan bits (Methods “Building an alphabet associating atomic signatures with Morgan bits”), we select 12 atomic signature candidates compatible with the input ECFP (Fig. 2c) (Methods “Signature-enumeration algorithm: atomic signature candidates”). The selection is performed by restricting the alphabet to tuples whose Morgan bit sets are subsets of the input ECFP and whose associated atomic signatures satisfy the bond matching conditions required by the consistency equations (Methods “Signature-enumeration algorithm: atomic signature candidates”). To obtain which combination of the atomic signature candidates could define molecular signatures, we compute the associated partition equations (PE) of the atomic signature candidates. These equations and their associated algebraic solving method are further explained in Methods “Signature-enumeration algorithm: integer partitions solving method”. The 18 partition equations (Fig. 2d) are solved independently using integer partitions, facilitated by Young tableaux (Fig. 2e). These solutions yield four distinct orbits (Fig. 2f), each corresponding to the common indices shared by the partition equations. Taking the product over the indices results in three partition equation solutions (Fig. 2g). Afterward, these solutions are subjected to 11 constraint (consistency and graphicality) equations (Fig. 2h) (Methods “Molecular signature”), yielding one final solution (Fig. 2i). A molecular signature composed of 11 atomic signatures is associated (Fig. 2j). Finally, to obtain the molecule(s) from the signature, the molecule-enumeration algorithm extracts possible atoms from atomic signatures and bond constraints from the molecular signature and then reconstructs a flat molecule (Fig. 2k) in 10 steps (Methods “Enumerating molecules from molecular signatures”). A stereoisomer-enumeration (Methods “Enumerating molecules from ECFPs”) is then applied to obtain the corresponding input molecule (Fig. 2l).

The results of the generative model applied to the same ECFP are given in Fig. 2m–o. The top 10 generative model predicts the ten most likely SMILES strings (Fig. 2m). Among these predictions, 8 are valid molecules (Fig. 2n), as verified by RDKit. The tenth generated molecule is a duplicate, while the fourth lacks stereochemical data. The similarity of each predicted molecule to the input ECFP is quantified using Tanimoto coefficients, which range from 1 to 0.538 (Fig. 2o). The first prediction, which is the top 1 prediction, corresponds to the target molecule and indicates the model's accuracy.

Molecules with numerous identical local environments can enumerate multiple distinct molecular structures and intermediate molecular signatures. Figure S4 illustrates this phenomenon using an eMolecules example, where the input ECFP enumerates 3 distinct molecular signatures and 6 unique molecules. When the generative model is applied to the same ECFP, the top 10 prediction produces 8 valid molecules but fails to generate any molecule that exactly matches the input ECFP.

### Generation vs. enumeration of molecules from ECFP on large datasets

In this section, we compare the deterministic enumeration and the generative model on large datasets to reconstruct molecular structures from ECFPs. To assess their efficiency, we use subsets of 10,000 molecules from the eMolecules and MetaNetX datasets (Methods “Datasets and sanitization”).

Table 1 shows the exceptionally high recovery rates of molecules from ECFPs using the enumeration algorithms, 99.66% (resp. 99.55%) for the MetaNetX (resp. eMolecules) database, using the MetaNetX (resp. eMolecules) alphabet (Methods “Building an alphabet associating atomic signatures with Morgan bits”). In principle our enumeration algorithms are guaranteed to produce all solutions, however, few cases can fail, as shown in Figure S5a-b, due to threshold settings designed to prevent excessively long computation times, which can occur in molecules with numerous cycles or many identical local environments. Table 2 summarizes the counts of enumerated molecules. For both test datasets, only 15 new intermediate molecular signatures were enumerated and a total of 21,291 (resp. 11,101) molecules have been enumerated for the MetaNetX (resp. eMolecules) test dataset. In particular, 175 (resp. 3) input ECFPs from the MetaNetX (resp. eMolecules) test dataset each gave rise to 10 or more distinct molecules, including 10 outliers whose input ECFPs generated from 57 up to 3,558 molecules (see Figure S5c–f). This observation was predominantly associated with molecules having numerous identical local environments, such as polycyclic structures (Fig. S5c–e) or nearly linear, acyclic compounds (Fig. S5f), with molecules from the MetaNetX database exhibiting these characteristics to a significantly greater extent than those from the eMolecules database. The capability to enumerate new molecules makes it useful for de novo molecular generation. The mean enumeration computation time was approximately 9.28 (resp. 8.5) seconds for the MetaNetX (resp. eMolecules) test dataset. We computed the Pearson correlation between the logarithm of the computation times for the deterministic enumeration and 30 different molecular complexity measures and descriptors, including the indices presented in Buehler et al. [51] (see Table S1). The highest correlation for both datasets was observed with the maximum component of the ECFP vector ($\rho =0.81$ for MetaNetX and $\rho =0.67$ for eMolecules). These results align with the fact that the enumeration procedure depends on integer partitions of the Morgan bit counts, whose number increases drastically as the integer value rises (Methods “Signature-enumeration algorithm: integer partitions solving method”).

**Table 1:** Percentage recovery using deterministic enumeration and percentage accuracy for the top 1, top 10 and top 100 generative outputs^a^

	MetaNetX	eMolecules
Recovery %: Enumerate signature from ECFP	99.92	99.64
Recovery %: Enumerate molecule from signature	99.74	99.91
Recovery %: Enumerate molecule from ECFP	99.66	99.55
Accuracy %: Generation top 1 molecule from ECFP	79.41	95.64
Accuracy %: Generation top 10 molecule from ECFP	91.57	99.23
Accuracy %: Generation top 100 molecule from ECFP	94.88	99.59

^a^Percentage recovery of query molecular signatures from ECFPs, and query molecules from molecular signatures and ECFPs using deterministic enumeration. Percentage accuracy for the top 1, top 10 and top 100 generated molecules from ECFPs using the generative model. All values are calculated on a test set of 10,000 molecules from the MetaNetX and eMolecules databases

**Table 2:** Comparison of the number of molecules enumerated by the deterministic algorithm versus those generated by the top 100 outputs of the generative model that match the input ECFP^a^

Database	Method	Count classes									
		0	1	2	3	4	5–9	10–14	15–19	20–24	25-inf
MetaNetX	Enumeration	20	8,111	1,131	35	300	228	67	42	34	32
	Generative	512	8,174	657	352	115	116	33	15	18	8
	% recovery		95.9	77.9	43.8	54.6	57	75.4	60.8	52.2	28.8
eMolecules	Enumeration	42	9,201	654	30	52	18		1		2
	Generative	41	9,288	460	173	15	21	1	1		
	% recovery		99.7	93.5	60	78.4	51.4		43.8		8.3

^a^These counts were computed for test sets of 10,000 ECFPs from the MetaNetX and eMolecules databases. The count classes are defined as follows: Class i represents the number of input ECFPs for which exactly i matching molecules were enumerated/generated. Similarly, Class i–j represents the number of input ECFPs for which between i and j (inclusive) matching molecules were enumerated/generated. The recovery rate for a given count class C is the percentage of enumerated molecules in class C that were also generated by the model, regardless of their count class in the generated set

Table 1 also presents the results about the top 1, top 10 and top 100 generated valid compounds from the Transformer model that match the input ECFPs. The percentage accuracies are highly encouraging, with 79.41% (resp. 95.64%) of predicted molecules having an ECFP matching the input vector in the top 1 position, 91.57% (resp. 99.23%) in the top 10 and 94.88% (resp. 99.59%) in the top 100 for the MetaNetX (resp. eMolecules) test dataset. The observed difference in accuracy between the test datasets can be attributed on the one hand to the smaller training set used for the transfer learning and, on the other hand, to the greater structural complexity of molecules in the MetaNetX database. Using RDKit to validate SMILES, we observed that 20.92% and 36.28% of the top 10 predicted strings were invalid molecules for the MetaNetX and eMolecules datasets, respectively. The proportion of invalid generated strings rose to 38.34% for MetaNetX and 51.94% for eMolecules among the top 100 predictions, underscoring the increasing difficulty of maintaining chemical validity as the number of predictions grows. The mean Tanimoto coefficient of valid molecules in the top 10 and top 100 predictions ranges from 0.70 to 0.80 for the MetaNetX and eMolecules datasets, reflecting a high degree of structural similarity between their ECFPs and the input fingerprints. A total of 9,488 and 9,959 molecules were generated within the top 100 predictions for the MetaNetX and eMolecules test datasets, respectively, highlighting differences in dataset complexity or diversity, as already noted using the deterministic enumeration. The mean generation computation time was approximately 0.44 s (resp. 0.56 s) for the MetaNetX (resp. eMolecules) test dataset in top 1 results, 0.88 s (resp. 0.96 s) in top 10 results and 6.2 s (resp. 6.52 s) in top 100 results. The Pearson correlation between the logarithm of the computation times and 30 different molecular complexity measures and descriptors was also computed for the generative model (see Table S1). Several descriptors related to molecular size and topology, including the number of atoms, number of bonds, molecular weight, the sum of the ECFP vector, the Harary index [52], and Proudfoot complexity [53], showed strong correlations ($\rho >0.8$) with computation time. The highest correlation for both datasets and across all top predictions was observed with the number of atoms (with $\rho$ ranging from $0.86 \,{\rm{to}} \,0.95$). These results are consistent with the principle of Transformer models working by iteratively expanding the sequence of tokens, where each token represents mainly atom, bonds and branching symbols.

The comparison between the number of enumerated molecules using the deterministic algorithm and those generated by the top 100 outputs of the generative model is given in Table 2. More than 95% of the molecules uniquely enumerated per ECFP have also been generated by the Transformer. However, in the highest count class, where more than 24 molecules are enumerated per ECFP, the generative model recovers only 28.8% and 8.3% of these structures for MetaNetX and eMolecules. The higher effectiveness at recovering molecules when the count of enumerated molecules is lower shows the difficulty for the model to generate all expected molecules from ECFPs of highly symmetric or linear molecules. This trend is further explained by the limitation of the fixed top 100 output size.

The test datasets, the enumerated and top 1, top 10 and top 100 generated molecules and the correlations between molecular complexity measures and computation times are given in Supporting Information (see “MetaNetX eMolecules DrugBank Enumeration Generation” file).

### Generation vs. enumeration of molecules from ECFP: comparison with the MolForge model

In the previous sections, we compared our deterministic enumeration algorithm to a state-of-the-art generative model conceptually similar to approaches like the MolForge work published in Ucak et al*.* [44]. Several differences exist between our experimental setting and that of MolForge, which required us to retrain a Transformer model with a slightly modified architecture, as outlined below:

- the ECFP used in MolForge is binary, while our approach requires a counted ECFP to satisfy the partition equations (PE), which connect locally generated Morgan bits with the complete molecular fingerprint;
- the stereochemistry parameter is set to its default False value during ECFP computation in MolForge, while it is explicitly considered in our approach;
- our work focuses on different regions of chemical space compared to MolForge.

We now compare accuracies of the deterministic enumeration, our generative model and the MolForge model on the MetaNetX, eMolecules and MolForge test datasets. Specifically, we processed the 10,000 MolForge test molecules following the procedure described in the method section “Datasets and sanitization” (omitting the molecular weight restriction), employed our own ECFP definition, and used a base alphabet derived from the MetaNetX, eMolecules, and MolForge datasets. Table 3 presents the recovery rates of deterministic enumeration and the accuracy of generative models (our model and the MolForge model) for reconstructing molecules from ECFP fingerprints across three datasets. Deterministic enumeration achieves near-complete recovery in all cases ($>97\%$), as expected from its design. The slightly lower recovery rate on the MolForge dataset is due to a number of timeouts, as this set includes larger molecules without a molecular weight restriction below 500 Daltons. Generative model performance varies across datasets and metrics. Both models show good accuracy when evaluated with binary fingerprints, where predicting the presence or absence of bits is less constrained than predicting exact counts. The MolForge model performs strongly on the eMolecules dataset as well as its native dataset. These results illustrate that inversing binary fingerprints is an easier task for generative models, and that performance is influenced by dataset composition and model training.

**Table 3:** Percentage recovery using deterministic enumeration and percentage accuracy for the top 1 generative outputs using the MolForge model and our model^a^

	MetaNetX	eMolecules	MolForge
Recovery %: Enumerate molecule from counted ECFP	99.66	99.55	97.39
Counted accuracy %: Generation top 1 molecule from counted ECFP using our model	79.41	95.64	62.07
Binary accuracy %: Generation top 1 molecule from counted ECFP using our model	85.93	97.81	70.99
Binary accuracy %: Generation top 1 molecule from binary ECFP using the MolForge model	49.64	91.3	87.16

^a^Percentage recovery of query molecules from ECFPs using deterministic enumeration. Percentage accuracy for the top 1 generated molecules from ECFPs using our generative model and the MolForge model. All values are calculated on 10,000 molecules from the MetaNetX, eMolecules and MolForge datasets

### Application of the deterministic enumeration: drug design

We now make use of the deterministic enumeration approach to identify novel analogues. To that end, we apply the enumeration algorithms to 9,516 ECFPs computed from 9,516 drug molecules derived from the version 5.1.12 of the DrugBank Online database [54] (Methods “Datasets and sanitization”). To achieve this, we computed and merged the alphabets of the MetaNetX, eMolecules, ChEMBL and DrugBank databases and obtained 1,119,246 unique tuples associating atomic signatures with ECFPs. The ChEMBL database was specifically included to improve coverage of drug-like chemical space and enhance the drug-likeness of the generated molecules. We then applied the deterministic enumeration algorithm on all these ECFPs with this specified alphabet and we obtained that 1,313 out of the 9,516 ECFP inputs generated a total of 3,691 new molecules. We screened PubChem to identify existing references for the newly enumerated molecules, finding that 21.08% (778 out of 3,691) were already referenced. Among these, 469 compounds have at least one associated patent, 217 have been tested in bioassays, and 170 exhibit at least one positive bioactivity result. The only non-single-atom-based molecules that generate new molecular signatures are estrone (DB00655), alcaftadine (DB06766), estrone sulfate (DB04574), paxalisib (DB15186), and emicerfont (DB12910) (Fig. S6a–e).

**Fig. 3:**
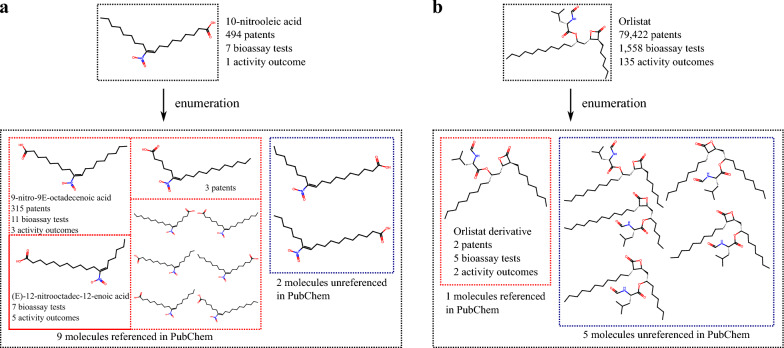
Examples of the enumeration algorithm applied to drug molecules. **a** The enumeration of 10-Nitrooleic acid (DB15026), or CXA-10, a small molecule drug in Phase II clinical trials (across all indications) with four investigational indications, resulted in eleven new molecules. Of these, nine are referenced in PubChem, while two are not. One of the PubChem-referenced molecules is associated with 315 patents and three positive bioassay results, including one shared assay with the parent drug [55]. One additional PubChem-referenced molecule yields 5 positive bioassay results, and another holds 3 associated patents. **b** The enumeration of Orlistat (DB01083), a reversible inhibitor of gastrointestinal lipases approved by the FDA for weight loss and weight maintenance, generated six new molecules. Among these, one is referenced in PubChem as an Orlistat derivative, with two patents and two positive bioassay outcomes [56], while the remaining five molecules are not referenced

To showcase our results, the enumeration of the 10-Nitrooleic acid (DB15026), a small-molecule drug currently in Phase II clinical trials with four investigational uses, is illustrated in Fig. 3a. Among the 11 newly enumerated molecules, 9 are listed in PubChem, while the remaining two are not. One of the PubChem-referenced molecules is associated with 315 patents and 3 positive bioassay results and two others also have 5 activity outcomes and 3 patents, respectively. As another example, the enumeration of the Orlistat drug, a reversible inhibitor of gastrointestinal lipases approved by the FDA for weight loss and maintenance, generated 6 new molecules and is illustrated in Fig. 3b. Among these, one is referenced in PubChem as an Orlistat derivative, with 2 patents and 2 positive bioassay outcomes, while the remaining 5 molecules are not referenced. Other examples of the deterministic enumeration applied on drug molecules are given in Figures S6, S7. Certain drugs yield a significant number of new molecules through the deterministic enumeration; in Table S2, we highlight 27 drug molecules whose ECFPs generate 20 or more new molecules.

The DrugBank database and the new molecules enumerated with their PubChem identifiers, the number of associated patents, bioassay tests and positive bioassay results are given in Supporting Information (see “MetaNetX eMolecules DrugBank Enumeration Generation” file). In Table S4 the bioassay test references for the enumerated molecules of Figs. 3, S6, S7 are provided.

## Discussion and conclusion

The study presents a comprehensive comparison between deterministic enumeration and generative models for reverse-engineering molecules from ECFPs. The findings highlight the strengths and limitations of both approaches in addressing the challenges of molecular design and reconstruction.

The deterministic enumeration algorithm uses a well-defined mathematical framework based on molecular signatures and linear Diophantine systems and ensures exhaustive exploration within the defined constraints, making it particularly suitable for applications requiring high precision, such as de novo drug design. The ability to generate novel molecular structures underscores its utility in exploring underrepresented areas of chemical space. Deterministic enumeration shows significant promise in drug discovery, as evidenced by their application to the DrugBank dataset, in the identification of novel molecules, including drug derivatives with potential therapeutic relevance, emphasizing its utility in systematic exploration. However, the computational intensity of the enumeration method is a notable limitation. The rapid growth of solution space, especially for highly symmetric molecules or those with complex topologies, necessitates the implementation of thresholds to mitigate computation times. This trade-off between completeness and efficiency could limit the method’s scalability for extremely large datasets or molecules with high complexity.

In contrast, the generative Transformer model offers a data-driven alternative for molecular reconstruction. This Transformer based generative model is conceptually similar to approaches like the MolForge work published in Ucak et al*.* [44]. However, it differs two important aspects, namely, it handles counted ECFP and operate on different regions of chemical space, allowing a direct comparison with our deterministic enumeration method. On the natural product dataset, the model achieves from nearly 80% matching of input ECFPs to predicted molecules in the top-1 predictions and over 94% in the top-100, showcasing its capability to provide relevant outputs even in challenging scenarios. Combining a comprehensive eMolecules dataset for pretraining with fine-tuning on a specialized natural product dataset nearly two orders of magnitude smaller achieves remarkably high accuracy. This two-step process bridges the gap between general chemical space and domain-specific molecular properties, allowing the model to generalize well while maintaining task-specific precision. This finding highlights the efficacy of transfer learning, particularly in contexts where access to large, specialized datasets is limited. Although its architecture is relatively simple, just 3 encoder- and 3 decoder-modules, our Transformer model delivers strong performance. This result demonstrates the potential of streamlined architectures when combined with carefully designed training strategies and datasets.

Deterministic enumeration takes an average of 9 s per molecule, whereas the generative model's runtime increases steeply with beam size, ranging from 0.5 s at beam size 1 to 6.36 s at beam size 100. This underscores the trade-off in generative modeling between accuracy and computational efficiency. The generative framework’s ability to extrapolate from training data to produce novel molecules adds a valuable dimension to de novo molecular design. However, generative models are not without limitations. Their inherent stochasticity and reliance on training data mean they are constrained by the quality and representativeness of their input datasets. This can lead to the prediction of invalid outputs, such as malformed or chemically nonsensical SMILES strings. Unlike deterministic methods, generative models do not guarantee exhaustive exploration, potentially overlooking certain valid molecular structures, as illustrated by the low recovery rates in Table 2 for high count classes of molecules enumerated by the deterministic method. In fact, the deterministic approach we propose can serve as a benchmark for generative methods, as demonstrated in this study using a Transformer architecture.

Future research should focus on improving the expandability and integration of deterministic methods, perhaps by optimizing partition algorithms or incorporating machine learning to guide the enumeration process. Similarly, enhancing the generative model’s capacity to handle underrepresented chemical spaces through advanced data augmentation techniques or transfer learning could address current limitations. The potential synergy between these approaches also warrants exploration. A hybrid framework that uses deterministic methods to define initial solution spaces, followed by generative models for refinement and property optimization, could offer a powerful tool for molecular design.

In both molecular and de novo drug design, controlling the diversity of generated structures is important for exploring novel regions of chemical space and moving beyond molecules closely related to known compounds. Within our framework, this can be achieved by using less structurally restrictive fingerprints, such as Functional-Class Fingerprints (FCFPs) [32], which describe atom environments based on functional roles rather than exact atomic identities. More generally, any fingerprint definition that satisfies the required partition equations can be used within our inversion framework.

ECFPs are already well-established molecular representation tools in chemistry, wildly used for QSAR/QSPR modeling, similarity searches, and virtual screening. In this paper, we demonstrate their applicability to chemical space exploration and de novo molecular and drug design, as the reverse-engineering techniques presented enable highly effective molecular reconstruction.

## Methods

### Datasets and sanitization

To train, define and compare the enumeration and the generative algorithms we consider two different databases. The first one is the July 2024 release of eMolecules [48] database which is a searchable platform of more than 18 million commercially available chemical compounds for use in medicinal chemistry, drug discovery, and chemical synthesis. The second database is the version 4.4 of the MetaNetX database, composed of around 200,000 molecule structures, that consolidates genome-scale metabolic networks and biochemical reactions, enabling the integration of metabolic models across multiple organisms and linking them to external biochemical and metabolic resources [47]. We sanitize these databases using the RDKit software [57] as follows. We select only molecules that are fully connected, have a molecular weight below 500 Daltons, and contain no wildcard atoms. While removing isotope information, we ensure stereochemistry is preserved. Additionally, for molecules with partially or completely missing stereochemical information, a stereoisomer is selected to complete the structure. After deduplication, we obtained 206,338 molecules for the MetaNetX database and randomly selected 5 million molecules from the eMolecules database. These molecules are represented using SMILES string notation [58] and are canonicalized with RDKit software. Special attention was given to ensure that molecules present in the MetaNetX dataset were not included in the eMolecules dataset.

For our drug design application, we utilized a dataset of drug molecules sourced from the version 5.1.12 of DrugBank Online [54], a freely accessible, comprehensive database integrating detailed information on drugs and their targets. This dataset consists of 9,516 drug molecules in SMILES format after sanitization, with no restriction on molecular weight applied in this case. To enhance the diversity of drug-like properties in the alphabet's molecular fragments, we incorporated bioactive, drug-like molecules from ChEMBL [59] release 35, which contains approximately 1.9 million molecules after sanitization. The PubChem database was downloaded locally to perform the screening of referenced molecules (accessed on 2024–08). This database comprises nearly 120 million molecules. The PubChem programmatic interface was used to determine bioassay results associated with a molecule (accessed on 2025–06-11), whereas patents were retrieved manually (accessed in June 2025). The code and datasets required to reproduce the results are publicly available online (see Associated Content).

### Extended-connectivity fingerprint

The Extended-Connectivity Fingerprint (ECFP) vector of a molecule, is computed using the Morgan algorithm [32, 38]. Initially, each atom in a molecule is assigned a unique identifier based on its atomic properties and with the help of a hashing function. The algorithm then iteratively updates these identifiers by considering the neighboring atoms within a specified radius $r$, effectively capturing the local molecular environments. The resulting vector encodes the presence of specific substructural patterns, making it valuable for tasks such as similarity searches, machine learning models for predicting molecular activity, and clustering molecules based on structural similarity. Let $nbits$ be a bitset size. For a molecule $m$ we denote by $ECFP(m)\in {\mathbb{N}}^{nbits}$ the counted ECFP of the molecule, that we calculate using the RDKit software and the GetMorganGenerator method [57]. The counted list of support indices of an ECFP are referred to as Morgan bits. In certain contexts, only the Morgan bits generated by a specific root atom and its local environments within a molecule are considered.

Unless stated otherwise, we use the convention $nbits=2048$ and $r=2$ throughout the paper. For use in the generative model and in the figures within this paper, ECFPs are also represented as character strings that list the Morgan bits, with each bit's position repeated according to its count.

### Molecular signature

We recall below the definition of a molecular signature and its basic properties [15, 17, 18]. Let $G=(V, E)$ be a molecular graph of a molecule $m$, let $x$ be one of its atoms that we call the root atom and let $r$ be a radius. The atomic signature ${\sigma }_{x}^{r}(G)$ is defined to be the subgraph of $G$ consisting of all atoms and bonds up to the radius $r$ from the atom $x$. Hence, the molecular signature ${\Sigma }_{G}^{r}$ of $m$ is defined to be the set of all atomic signatures appearing in the molecular graph $G$. Note that stereochemistry information is excluded from these subgraphs, as breaking the molecule into smaller fragments can occasionally disrupt chirality. We use the SMARTS notation for atomic and molecular signature fragments [16]. For simplicity, we have chosen to represent atomic signatures as rooted graphs in the figures. We now give two constraint properties satisfied by molecular signatures that will be useful later on:

- Consistency equations (CE) ensure interdependence among the atomic signatures of a molecular signature in the sense that any bond connecting radius $r-1$ atomic signatures in a radius $r$ atomic signature will match up with a bond in another atomic signature in reverse order:
  $$\#({\sigma }_{x}^{\text{r}-1}\to {\sigma }_{y}^{\text{r}-1})=\#\left({\sigma }_{y}^{\text{r}-1}\to {\sigma }_{x}^{\text{r}-1}\right),$$
  $$\#({\sigma }_{x}^{\text{r}-1}\to {\sigma }_{x}^{\text{r}-1})\in 2{\mathbb{N}},$$
  CE $$\forall {\sigma }_{x}^{\text{r}-1}, {\sigma }_{y}^{\text{r}-1}\in {\Sigma }_{G}^{\text{r}-1}.$$
- Graphicality equation (GE) ensures that at least one connected graph can be constructed from the molecular signature. Let ${\Sigma }_{G}^{0}$ be the radius $0$ molecular signature extracted from the molecular signature ${\Sigma }_{G}^{r}$ as the collection of the radius $0$ subgraphs ${\sigma }_{x}^{0}$ of the radius $r$ atomic signature graphs ${\sigma }_{x}^{r}$. Let $N=\left\{{n}_{1}, ... , {n}_{k}\right\}$ be the sequence of integers where ${n}_{i}$ is the number of vertices of degree $i$ in ${\Sigma }_{G}^{0}$. Thus, to construct at least one connected graph from the molecular signature, the following equation must be satisfied:
  GE $${\sum }_{i=2}^{k}(i-2){n}_{i}-{n}_{1}+2\in 2{\mathbb{N}}.$$

Finally, for each atom $x$ of $G$, we denote by $ECFP({\sigma }_{x}^{r})$ the set of the Morgan bits in the ECFP generated by $x$ up to the radius $r$. The compatibility between the ECFP of the molecule $m$ and its atomic signatures reads as partition equations (PE) that are $nbits$-equations defined by the fact that the sum of the Morgan bits of the atomic signatures is equal to the ECFP:

PE $${\sum }_{{\sigma }^{r}\in {\Sigma }_{G}}ECFP\left({\sigma }^{r}\right)=ECFP\left(m\right).$$

As for the ECFP, unless stated otherwise, we use the convention $r=2$ throughout the paper.

### Building an alphabet associating atomic signatures with Morgan bits

A key component of our deterministic approach to reconstruct molecules from an ECFP is what we define as an alphabet, which is a set that establishes a correspondence between atomic signatures and Morgan bits. Given a database of molecules $D$ and a molecule $m\in D$, we compute, for each atom $x$ in $m$, a tuple consisting of the set of Morgan bits generated by the atom $x$ in the molecular graph $G$ of $m$ (up to the radius $r$) and the radius $r$ atomic signature ${\sigma }_{x}^{r}$. Repeating this process for all molecules in the database $D$, we construct the alphabet $\mathcal{A}$ of $D$. More precisely:

$$\mathcal{A}=\left\{(ECFP({\sigma }_{x}^{r}),{\sigma }_{x}^{r}) |\forall {\sigma }_{x}^{r}\in {\Sigma }_{G}^{r}, \forall G\in D\right\}.$$

### Enumerating molecular signatures from ECFPs

In this section, we explain the deterministic method that associates molecular signatures from an ECFP input vector by solving a Diophantine system defined on a pre-calculated alphabet of atomic signatures.

While general structural results have been proven on the set of positive integer solutions of a Diophantine equation  [60], so far only greedy methods exist to explicitly obtain all the positive integer solutions of a general linear Diophantine system [61, 62]. In our case, the linear Diophantine system has the particularity that the resolution of a subset of its equations can be interpreted as finding integer partitions, which is a well-known problem in number theory that can be solved by computing Young tableaux of integers [63]. The partition function $p(n)$, which counts the number of distinct ways to express an integer $n$ as a sum of positive integers, must be calculated through recurrence relations, as no closed-form expression exists, only asymptotic approximations. This function reveals intriguing mathematical properties [63], such as Euler’s pentagonal number theorem and Ramanujan's modular congruences, and is fundamental in number theory and combinatorics, with applications extending from statistical mechanics to modular forms.

The main steps of the signature-enumeration are as follows:

- From a database of molecules, we construct an alphabet $\mathcal{A}$ associating atomic signatures to Morgan bits.
- Given an ECFP, we define a linear Diophantine system with consistency, graphicality and partition equations on tuples from the alphabet of atomic signatures associated with Morgan bits.
- We select atomic signature candidates from the alphabet that could potentially define molecular signatures satisfying the linear Diophantine system.
- Finally, we solve this system using integer partitions and action groups and we obtain molecular signatures corresponding to the input ECFP.

We detail below all the parts of the signature-enumeration algorithm.

### Signature-enumeration algorithm: Diophantine system

Let $ECFP(m)$ be an ECFP. Firstly, the sets of atomic signatures from the alphabet that define molecular signatures must satisfy the (CE) and (GE) equations. Furthermore, such a molecular signature can then be associated with a molecule having the input $ECFP(m)$ as its ECFP representation only if it also satisfies the (PE) equations. Hence, if we denote the elements of the alphabet $\mathcal{A}$ by ${A}_{1}, ... ,{A}_{l}$ and rewrite these equations as matrix equations using the basis $\left\{{A}_{1}, ... ,{A}_{l}\right\}$, then the problem is reduced to solve the positive integer solutions of the linear Diophantine system defined by the (CE, GE, PE) equations. That it so that, we have to compute:

$$\mathcal{S}=\{ \left({n}_{j}\right)\in {\mathbb{N}}^{l} | \left({n}_{j}\right) \text{satisfies to} (CE), (GE) \ (PE)\}$$

In general, the alphabet is a very large set and hence the linear Diophantine system can have a considerable shape. To facilitate solving calculations, it is possible to restrict the alphabet to a minimal set that we call atomic signature candidates, therefore reducing the system size.

### Signature-enumeration algorithm: atomic signature candidates

To satisfy to the partition equations (PE) and since the values in ECFP vectors are always positive, we can, without loss of generality, restrict our alphabet to tuples $\left(ECFP\left({\sigma }_{x}^{r}\right),{\sigma }_{x}^{r}\right)$ where the ECFP vector is less than or equal to the ECFP input vector. That is to say, we restrict the alphabet $\mathcal{A}$ to the set $\mathcal{A}\mathcal{^{\prime}}$ defined by:

$$A^{\prime} = \left\{ {(ECFP(\sigma_{x}^{r} ),\sigma_{x}^{r}) \in A|ECFP(\sigma_{x}^{r} ) \le ECFP(m)} \right\}.$$

On the other hand, an atomic signature ${\sigma }_{x}^{r}$ could only contribute to satisfy to the consistency equations (CE) if the bond ${\sigma }_{x}^{r-1}\to {\sigma }_{y}^{r-1}$ that connects the subgraphs ${\sigma }_{x}^{r-1}, {\sigma }_{y}^{r-1}$ of radius $r-1$ for any ${\sigma }_{y}^{r-1}\subseteq { \sigma }_{x}^{r}$ appears as in reverse order in any other atomic signature. Hence, without loss of generality, we can restrict $\mathcal{A}\mathcal{^{\prime}}$ to its maximal subset $\mathcal{A}\mathcal{^{\prime}}\mathcal{^{\prime}}$ verifying this condition. Since the union of any two subsets satisfying this condition also satisfies it, then the maximal subset $\mathcal{A}\mathcal{^{\prime}}\mathcal{^{\prime}}$ is well-defined, and we refer to its elements as the atomic signature candidates. Hence, solving the system on the alphabet $\mathcal{A}$ is equivalent to solving the system on its subset $\mathcal{A}\mathcal{^{\prime}}\mathcal{^{\prime}}$ which simplifies calculations. By abuse of notation, we also denote elements of $\mathcal{A}\mathcal{^{\prime}}\mathcal{^{\prime}}$ by $\left\{{A}_{1}, ... ,{A}_{l}\right\}$ and we call them the atomic signature candidates.

### Signature-enumeration algorithm: integer partitions solving method

The linear Diophantine system has the particularity that solving the ith partition equation is equivalent to find integer partitions of the Morgan counted bit ${ECFP(m)}_{i}$ appearing in the ECFP vector at the index $i$ into a sum of the Morgan bits ${ECFP\left({\sigma }_{x}^{r}\right)}_{i}$ of the atomic signature candidates $(ECFP({\sigma }_{x}^{r}),{\sigma }_{x}^{r})$ selected from $\mathcal{A}\mathcal{^{\prime}}\mathcal{^{\prime}}$. The set of solutions of the partition equations is denoted by $\mathcal{S}(PE)$ and calculated by the following algorithm.

We first compute independently the solutions for each partition equation:

$$\mathcal{S}\left(PE,i\right)=\left\{\left({n}_{\text{j}}\right)\in {\mathbb{N}}^{l} |{\sum }_{j=1}^{l}{n}_{j}{ECFP\left({A}_{j}\right)}_{i}={ECFP\left(m\right)}_{i}\right\}$$

for all $i$ such that $1\le i\le nbits$ and ${ECFP\left(m\right)}_{i}\ne 0$. We have

$$\mathcal{S}\left(PE,i\right)=\mathcal{S}\left(PE,i,>\right)+Ker\left(PE,i\right),$$

where.

$$\mathcal{S}\left(PE,i,\right)=\left\{\left({n}_{\text{j}}\right)\in {\mathbb{N}}^{l} \right| {\sum }_{j=1}^{l}{n}_{j}{ECFP\left({A}_{j}\right)}_{i}={ECFP\left(m\right)}_{i} {n}_{j}=0\,\, \text{if}\,\, {ECFP\left({A}_{j}\right)}_{i}=0\},$$

$$Ker\left(PE,i\right)=\left\{\left({n}_{j}\right)\in {\mathbb{N}}^{l} | {n}_{j}=0\,\, \text{if}\,\, {ECFP\left({A}_{j}\right)}_{i}\ne 0\right\}.$$

Hence, computing the elements of $\mathcal{S}(PE,i,>)$ is equivalent to calculate the partitions of the non-zero integer ${ECFP\left(m\right)}_{i}$ into non-zero integers $\left\{{ECFP\left({A}_{j}\right)}_{i} | \forall {A}_{j}\,\, \text{s.t.}\,{ECFP\left({A}_{j}\right)}_{i}\ne 0\right\}$. This well-known problem has been extensively studied in number theory and it turns out that integer partitions can be obtained via Young tableaux [63] and computed through recurrence relations. For any integer partition $\left({\lambda }_{1}\ge ... \ge {\lambda }_{n}\right)$ obtained in this way, we have to consider permutations $\sigma \cdot \left({\lambda }_{1}, ... ,{\lambda }_{n}\right)$, for $\sigma$ in the permutation group ${Sym}_{n}$ , and complete them by adding zeros at indices $j$ where ${ECFP\left({A}_{j}\right)}_{i}=0$. In this way, $\mathcal{S}(PE,i,>)$ is obtained and consequently $\mathcal{S}(PE,i)$ is also obtained. We notice that, rarely, the non-zero positive integer ${ECFP\left({A}_{j}\right)}_{i}$ is not equal to 1, since an atom can locally create several times the same Morgan bit, and so in that case we have to not take all the partitions and permutations.

In general, an atomic signature can contribute to several Morgan bits, and hence the sets $\mathcal{S}(PE,i,>)$ have to be carefully combined to obtain the solution set $\mathcal{S}(PE)$. To that end, we use group theory [64] to see which sets $\mathcal{S}(PE,i,>)$ are related. We consider the permutation group ${Sym}_{l}$ and define $H$ to be its subgroup generated by all the transpositions.

$$\left\{(j,k) | \exists i \,\,\text{s.t.}\, {ECFP\left({A}_{j}\right)}_{i}{ECFP\left({A}_{k}\right)}_{i}\ne 0\right\}.$$

The group action of $H$ can be used to decompose $\left\{1, ... , l\right\}$ into a sum of orbits $\left\{1, ... , l\right\}={\cup }_{k}O(k)$ where $k\in \left\{1, ... , l\right\} / H$. For ${i}_{1},{i}_{2}\in O(k)$, the combined solution set is given by

$$\left\{\text{max}\left(u,v\right) \right| \forall u\in \mathcal{S}\left(PE,{i}_{1},\right),\forall v\in \mathcal{S}\left(PE,{i}_{2},\right) \,s.t.\,\, u,v\,\, \text{are compatible}\}$$

where $u, v$ are said to be compatible if ${u}_{i}={v}_{i}$ for all $i$ such that ${ECFP\left({A}_{i}\right)}_{{i}_{1}}{ECFP\left({A}_{i}\right)}_{{i}_{2}}\ne 0.$ By repeating iteratively this process for all indices of $O(k)$, we obtain the orbit solution set $O\mathcal{S}(PE,k,>)$ associated to the orbit $O(k)$. Finally, we have to combine solutions of distinct orbit solution sets. For $O\mathcal{S}(PE,{k}_{1},>)$ and $O\mathcal{S}(PE,{k}_{2},>)$ the associated set of solutions is given by

$$\left\{max(u,v) | \forall u\in O\mathcal{S}(PE,{k}_{1},>),\forall v\in O\mathcal{S}(PE,{k}_{2},>)\right\}$$

where no compatibility check is required since vectors are from distinct orbits. By repeating iteratively this process for all orbits $O(k)$ we then obtain the partition equation solutions $\mathcal{S}(PE)$. Remark that we have

$$\left|\mathcal{S}(PE)\right|={\prod }_{k}\left|O\mathcal{S}(PE,k,>)\right|.$$

To get all the solutions we restrict the solution vectors of $\mathcal{S}(PE)$ to the consistency and graphicality equations:

$$\mathcal{S}=\left\{\mathcal{X}\in \mathcal{S}(PE)|\mathcal{X} \text{satisfies to} (CE) \& (GE)\right\}.$$

### Signature-enumeration algorithm: partition threshold settings

The number of integer partitions increases significantly as the Morgan bit ${ECFP\left(m\right)}_{i}$ value rises. For example $p(20)=627$ and the cardinality of their associated permuted vectors is $\text{92,378}$. To prevent excessively long computation times, we have set a limit of ${T/10}^{2}$ on the number of integer partitions per equation and $T$ for the number of associated permutations of each partition. We observed that, generally, solutions to equations with smaller norms are more likely to satisfy other integer partition equations compared to those with higher norms. Consequently, when restricting the number of integer partitions considered, we prioritize selecting the vectors with the smallest norms. Unless stated otherwise, we use the convention $T=2\times {10}^{5}$ throughout the paper.

### Enumerating molecules from molecular signatures

In this section we explain the deterministic method to obtain molecular graphs from a molecular signature. The molecule-enumeration algorithm, coming from Faulon et al*.* [17], consists of extracting atoms and bond constraints from atomic signatures and then recursively reconstructs the possible molecular structures. Here are the main steps of this algorithm.

We begin by extracting all the atoms and their properties, such as atomic number and formal charge, from the atomic signatures of the molecular signature, in order to initialize a disconnected graph G, meaning it is edgeless. Then, for every atomic signature ${\Sigma }_{G}^{r}$ we extract constraints from the consistency equations (CE) describing the bond(s) of the root atom ${\sigma }_{x}^{r-1}$ to its neighbor atom(s) ${\sigma }_{y}^{r-1}$. Hence, for each pair of nodes $x, y$ of the graph $G$, having respectively ${\sigma }_{x}^{r-1}, {\sigma }_{y}^{r-1}$ as $r-1$ atomic signatures, this gives the possible bonds between them. Finally, a recursive algorithm along all the possible connections between atoms is used to enumerate all the connected molecular graphs that can be constructed with these constraints. We then select only the molecular graphs having the input molecular signature as molecular signature.

To prevent excessively long computation times, we have imposed a threshold limit $T$ on the number of steps utilized by the algorithm. If this threshold is reached, we repeat the process up to 10 times, randomly selecting untested possible molecular combinations. As for partition threshold settings, unless stated otherwise, we use the convention $T=2\times {10}^{5}$ throughout the paper.

### Enumerating molecules from ECFPs

Let $ECFP(m)$ be an ECFP that we want to reverse-engineer. From an alphabet $\mathcal{A}$ constructed from a database of molecules $D$, we apply the signature-enumeration algorithm and we obtain a set of molecular signatures $\mathcal{S}$ from $ECFP(m)$. Then, we apply the molecule-enumeration algorithm on each molecular signature ${\Sigma }_{G}\in$ $\mathcal{S}$ to obtain a set of molecules $\mathcal{M}$. For each non-stereospecific molecule $m\in \mathcal{M}$, we compute its stereoisomers using the EnumerateStereoisomers function from RDKit [57]. To prevent excessively long computation times, we have imposed a limit of ${2\times 10}^{5}/|\mathcal{M}|$ isomers enumerated per molecule in $\mathcal{M}$. We finally select all of these molecules having the same ECFP representation as the input ECFP vector $ECFP(m)$.

### Generative model to predict SMILES from ECFPs

The method employs a data-driven approach using a Transformer architecture to predict SMILES strings from ECFPs. Transformer models are deep learning architectures that have revolutionized natural language processing and sequence modeling due to their ability to capture complex dependencies within sequences. They operate based on a self-attention mechanism, allowing the model to weigh the importance of different parts of a sequence in parallel, rather than sequentially, as in recurrent models. This enables Transformers to handle long-range dependencies efficiently and learn intricate patterns in data.

**Fig. 4:**
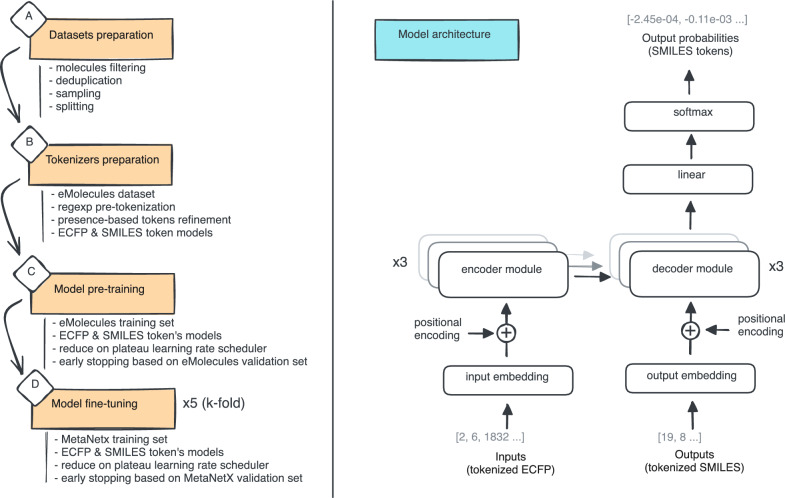
Summary of the pipeline for training the generative model (left) and architecture of the Transformer model (right). The pipeline consists of the following steps: **a** data is sourced from the MetaNetX and eMolecules datasets, as detailed in the Methods section. These datasets are processed to create training, validation, and test subsets for both eMolecules and MetaNetX. **b** eMolecules is used to construct SentencePiece tokenizers, which convert ECFP and SMILES representations into integer vectors ready for use. **c** A pre-trained model is developed using the eMolecules training and validation datasets. **d** Five fine-tuned models are subsequently built, one for each of the five folds in the MetaNetX dataset. The Transformer model is illustrated using a classical representation inspired by Vaswani et al*.* [45]. The primary difference lies in the use of only three encoder and decoder modules, instead of the standard six

In the context of our study, the Transformer model is adapted to treat the ECFP vectors as input features and the SMILES strings as target sequences. ECFPs, which are fixed-length vectors, serve as the input embedding for the Transformer. The model is trained to map these high-dimensional ECFP vectors to their corresponding SMILES representations. After training, the model can predict novel SMILES strings from unseen ECFP inputs, offering a generative approach to molecule reconstruction based on molecular fingerprints. Figure 4 gives an overview of the pipeline employed.

#### eMolecules dataset

The 5 million molecules sampled from the eMolecules database were used. This dataset was further divided into training, validation, and test subsets with an 80/10/10 split ratio. Distributions of atom types and molecular properties were investigated to ensure a good spread of individuals and representativeness of the dataset (Fig. S8).

#### MetaNetX dataset

The 206,338 molecules sanitized from MetaNetX database were utilized. A test set comprising 10% of these molecules was built, and the remaining 90% was divided into five folds for cross-validation, leading to five pairs of training and validation subsets. Distributions of atom types and molecular properties were analysed to ensure a good spread of individuals and dataset representativeness (Fig. S8).

#### Tokenization

Token lists were constructed using a word-based algorithm applied to the eMolecules dataset. For SMILES strings, atom-wise and bond-wise decomposition was performed to break them into meaningful tokens. Specifically, SMILES strings were tokenized using the regular expression from Schwaller et al. [65], effectively capturing the chemical syntax by identifying atoms, bonds, rings, and branches. The regular expressions are “(\[[^\]]+]|Br?|Cl?|N|O|S|P|F| I|b|c|n|o|s|p|\(|\)|\.|=|#|-|\ +|\\\\|\/|:|~|@|\?|>|\*|\$|\%[ 0-9]{2}|[0-9])”. ECFPs were pre-tokenized by retaining only the representation in Morgan bits (bits set to 1 or more), which correspond to the presence of specific substructures within the molecule. To reduce the vocabulary size and focus on the most significant features, tokens that appeared in less than 0.01% of molecules were disregarded (see Table S3). Additionally, four special tokens representing start-of-sequence (< SOS >), end-of-sequence (< EOS >), padding (< PAD >), and unknown tokens (< UNK >) were added. This tokenization process resulted in vocabulary sizes of 2,052 tokens for ECFP vectors and 47 tokens for SMILES strings.

#### Model architecture

A minimized implementation of the Transformer model introduced in the seminal paper of Vaswani et al. [45] was developed using PyTorch and Lightning libraries (Fig. 4). The Transformer architecture is composed of 3 encoder- and 3 decoder-modules, each module was composed of 8 attention heads, the model dimension was 512, and the feed-forward layers have a dimensionality of 2,048. This configuration balances model complexity with computational efficiency.

#### Model training

The training process was divided in two stages: pre-training and fine-tuning. For both stages, the AdamW optimiser algorithm [66] was employed, with an initial learning rate set to 10^−4^ and a weight decay coefficient of 10^−2^. To adjust the learning rate dynamically during training; the ReduceLROnPlateau scheduler [67] was utilized, enabling the learning rate to decrease adaptively over epochs based on the accuracy metric. An effective batch size of 128 was used, achieved when required through gradient accumulation across multiple steps to accommodate hardware constraints.

Training was conducted on NVIDIA GPUs with 40 GB of memory per device. The entire training process required approximately 24 h for pre-training and 2 h for fine-tuning. To promote transparency and reproducibility, the source code, along with detailed instructions for running experiments, is publicly available on GitHub (see “Supporting Information”). Additionally, all hyperparameter configurations and data preprocessing scripts have been included in the repository to ensure ease of replication.

Per stage specific set up:

- Pre-training: The Transformer model was initially trained using the eMolecules train dataset for 42 epochs. The validation dataset was used to monitor the model’s accuracy, and training was halted when the accuracy did not improve by at least 0.01% after five consecutive epochs.
- Fine-tuning: The pre-trained model was fine-tuned using the MetaNetX dataset to adapt the model to molecules relevant to metabolic networks. During fine-tuning, the encoder modules were frozen, focusing the refinement on the decoder’s capabilities towards MetaNetX-like molecules. Five fine-tuned models were generated from each of the five-fold training datasets. Validation datasets were used to monitor accuracy, and training was stopped when the accuracy did not improve by at least 0.01% after six consecutive epochs.

**Model Evaluation:** Both the pre-trained (from eMolecules) and fine-tuned (from MetaNetX) models were evaluated using their respective test sets, ensuring that tested molecules were not present in the training or validation datasets. Evaluation metrics included string equality, whether the predicted SMILES strings are strictly equals to the expected one, and Tanimoto Similarity, assessing with the Tanimoto coefficient the similarity between ECFP computed from the predicted SMILES and ECFP used as input. All molecular manipulations and computations were performed using RDKit’s version 2024.09.3 [57].

## Associated content

### Supporting information

The supplementary information contains additional figures and tables related to the distributions of the molecular databases and the enumeration and generative algorithms:

Additional figures and tables (PDF).

The supplementary information also contains the results of the enumeration and the Transformer algorithms on the test datasets derived from the MetaNetX and eMolecules datasets as well as the results of the enumeration algorithm on the DrugBank molecules:

MetaNetX eMolecules DrugBank Enumeration Generation (Excel).

## Supplementary Information

Additional file 1.

Additional file 2.

## Data Availability

The code associated to the molecular signature and enumeration algorithms is available at: 10.5281/zenodo.15682681. The code associated to the Transformer model and the paper's results is available at: 10.5281/zenodo.15689213. The datasets and weights of the fitted Transformer model are available at: 10.5281/zenodo.15682264. Two GitHub repositories are associated to this article: The molecule-signature repository containing the package to compute molecular signatures and to perform molecular enumeration from ECFPs: https://github.com/brsynth/molecule-signature. The molecule-signature-paper repository containing the code related to the Transformer model and the paper's results: https://github.com/brsynth/molecule-signature-paper.

## Abbreviations

ECFP: Extended-connectivity fingerprint
QSAR: Quantitative Structure–Activity Relationship
QSPR: Quantitative Structure–Property Relationship
RNN: Recurrent neural network
LSTM: Long short-term memory network
CE: Consistency equation
PE: Partition equation
GE: Graphicality equation
