# Supplementary material for: Reverse engineering molecules from fingerprints through deterministic enumeration and generative models
Source: J Cheminform. 2025 Oct 15;17:157. doi: 10.1186/s13321-025-01074-5 (PMC12522240; doi:10.1186/s13321-025-01074-5)
Supplement: Supplementary file 2 — Additional file 2. [file 13321_2025_1074_MOESM2_ESM.docx]

Supporting information for

**Reverse Engineering Molecules from Fingerprints through Deterministic Enumeration and Generative Models**

Philippe Meyer^1^, Thomas Duigou^1^, Guillaume Gricourt^1^, Jean-Loup Faulon^1,2,*^

1: Université Paris-Saclay, INRAE, AgroParisTech, Micalis Institute, 78350, Jouy-en-Josas, France

2: The University of Manchester, Manchester Institute of Biotechnology, Manchester M1 7DN, U.K.

*: corresponding author

**Table of Contents**

**Figure S1. Number of molecules sharing the same molecular signature and ECFP, analyzed across varying radiiS2**

**Figure S2. TMAP visualization of chemical space coverage for three molecular databases: MetaNetX, eMolecules and ChEMBLS3**

**Figure S3. Comparison of the MetaNetX, eMolecules and ChEMBL datasets and evolution of the percentage of molecules introducing new elements into the alphabet as a function of the number of molecules processed from the eMolecules database. S4**

**Figure S4. Example of the deterministic enumeration of an ECFP that generates multiple molecular signatures and molecules and where the generative model fails to produce a molecule with the correct ECFP S5**

**Figure S5. Molecules not reconstructed by the deterministic enumeration algorithm from their ECFPs due to threshold settings imposed to limit computation times and molecules from the MetaNetX database that produce a lot of new molecules through the deterministic enumeration S6**

**Figure S6. Drug molecules that enumerate new molecules via novel molecular signatures as intermediates and deterministic enumeration algorithm applied on the Mitoxantrone, Linoleic acid and Silybin molecules S8**

**Figure S7. Deterministic enumeration algorithm applied on the CUCDC-101, Cycrimine, Valdecoxib and Pimavanserin molecules S8**

**Figure S8. Comparative distributions of molecular properties across eMolecules and MetaNetX subsets S9**

**Table S1. Pearson correlation between the logarithm of the computation times for the enumeration and generation methods and 30 different molecular complexity measures and descriptors. S10**

**Table S2. Drug molecules enumerating 20 or more new molecules with identical ECFP S11**

**Table S3. Statistics on tokens extracted from eMolecules dataset S13**

**Table S4. Bioassay test references for enumerated molecules of example drug molecules S14**


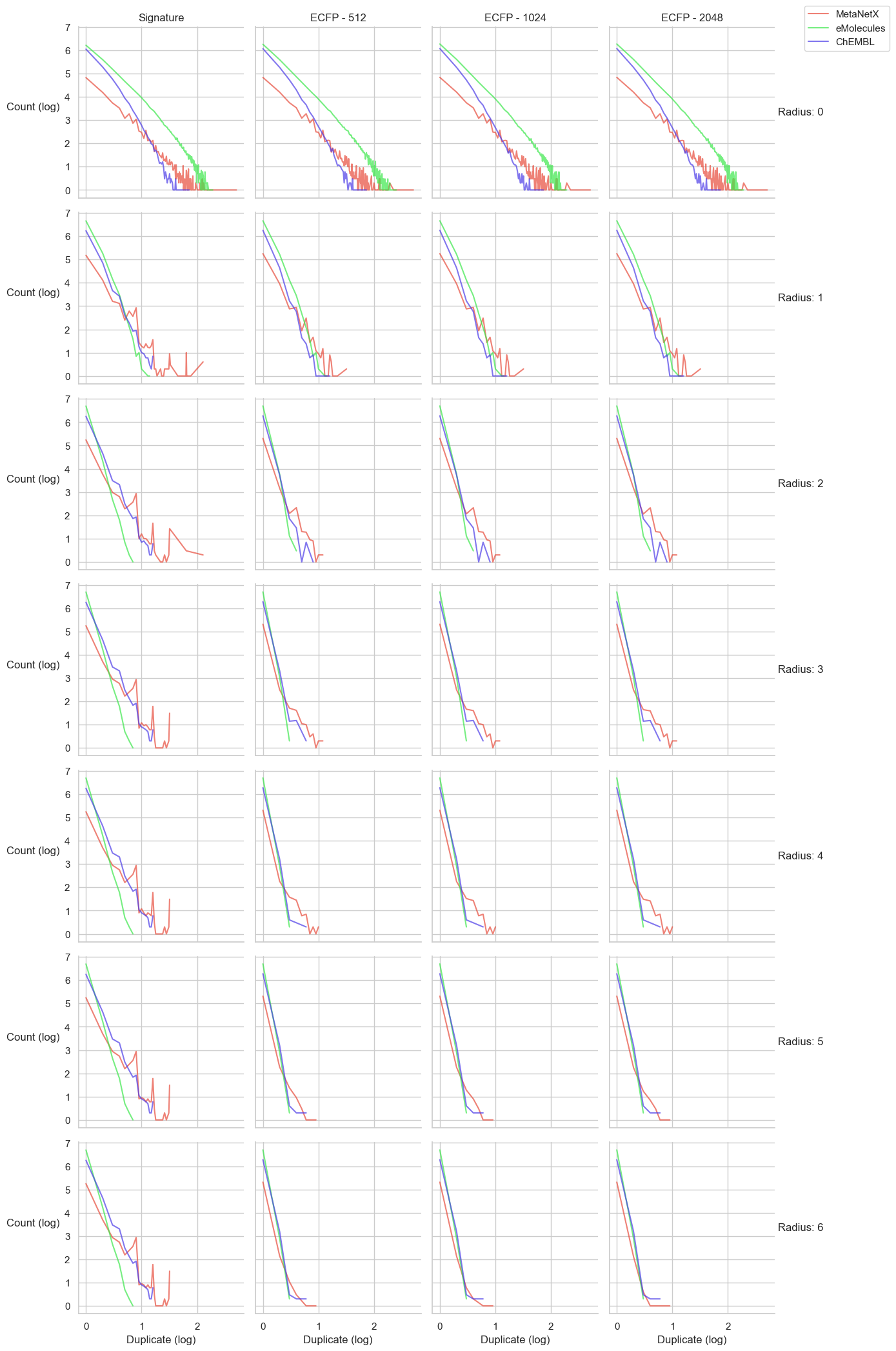


Figure S1. Number of molecules sharing the same molecular signature and ECFP, analyzed across varying radii. The counts were conducted for molecular signatures and ECFP using 512, 1024, and 2048 bits, based on data from MetaNetX, eMolecules and ChEMBL at radii levels 0 to 6. The graphs show a decreasing number of duplicates as the radii increase. Furthermore, no significant trend differences are observed between ECFP representations with 512, 1024, and 2048 bits.


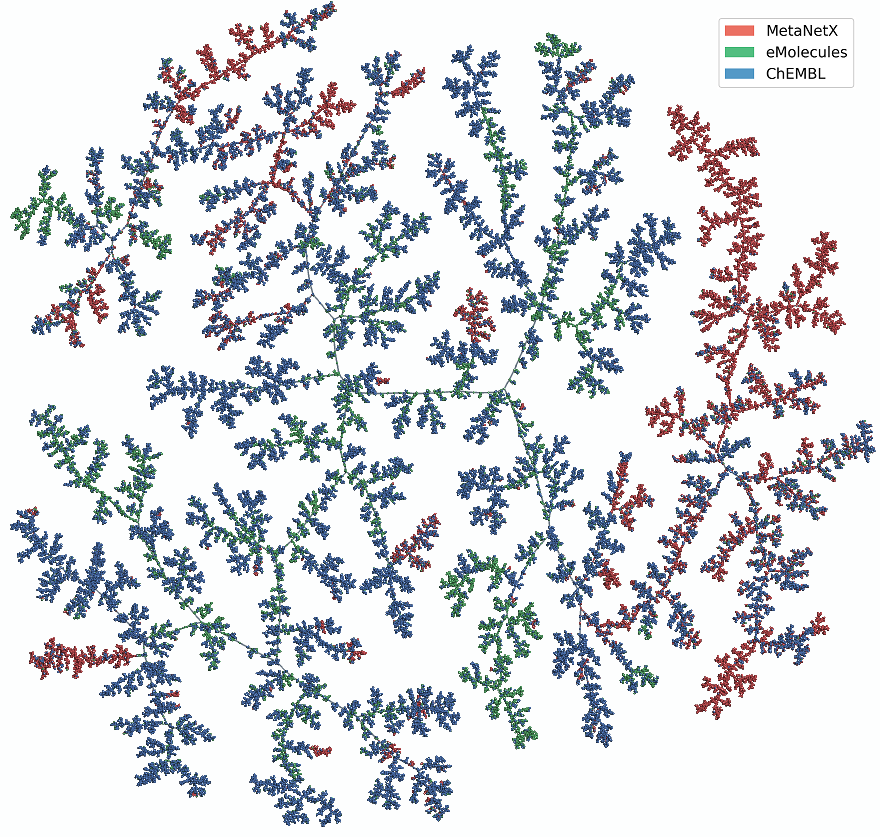


Figure S2. TMAP visualization of chemical space coverage for three molecular databases: MetaNetX, eMolecules and ChEMBL. For each database, 200,000 molecules were randomly sampled and encoded into molecular fingerprints for visualization. The resulting map reveals distinct structural clusters and partially overlapping regions, indicating both shared and unique chemical subspaces across the datasets. Notably, the main backbone of the map is predominantly shaped by ChEMBL molecules (blue), forming the core structure of the chemical space. In contrast, MetaNetX (red) and eMolecules (green) contribute additional branches and localized clusters, highlighting their more specialized chemical coverage.


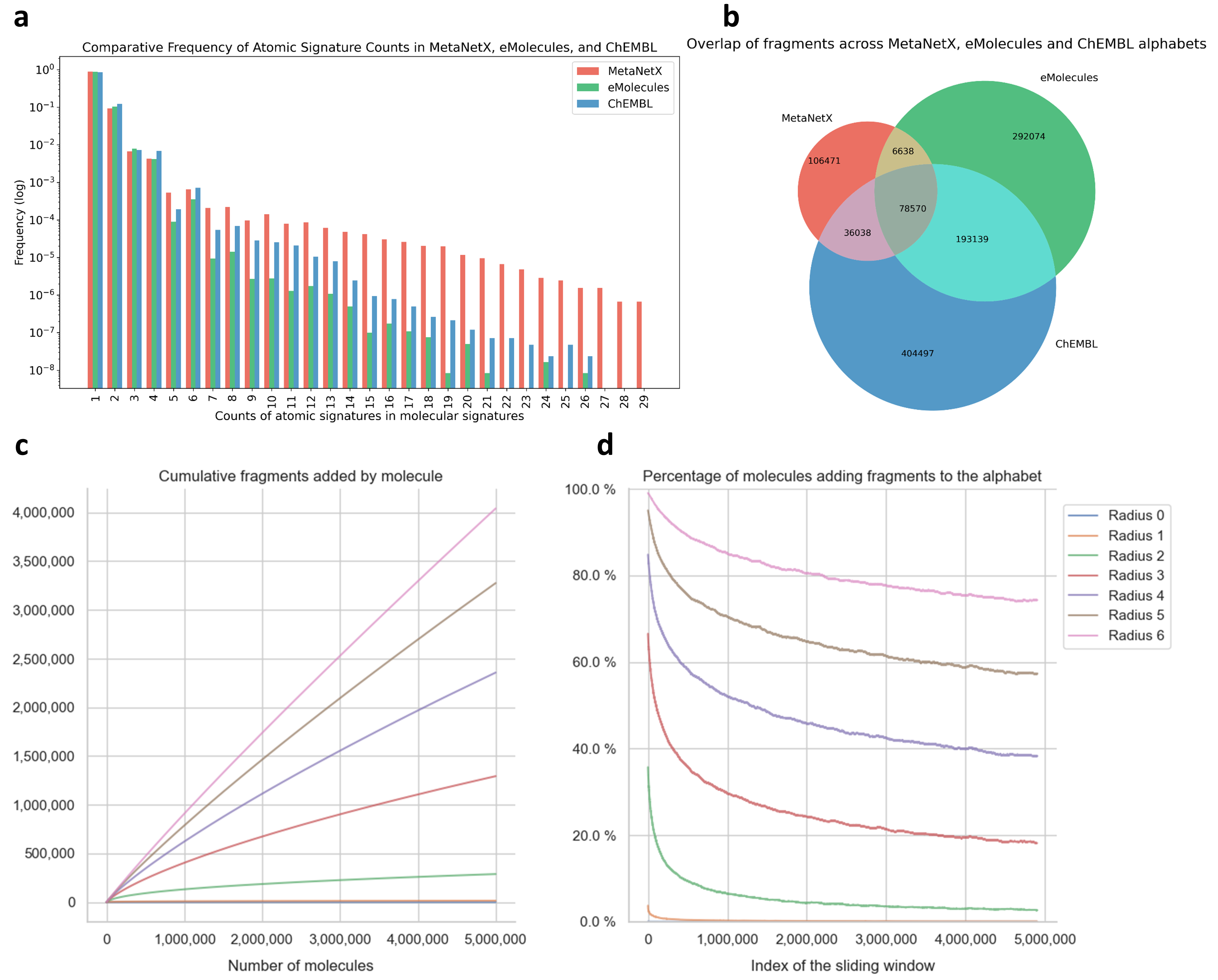


Figure S3. Comparison of the MetaNetX, eMolecules and ChEMBL datasets and evolution of the percentage of molecules introducing new elements into the alphabet as a function of the number of molecules processed from the eMolecules database. (a) Counting the frequencies of repetitions of atomic signatures in molecular signatures shows that the molecules from the MetaNetX database have more identical local environments than molecules in the eMolecules and ChEMBL databases. (b) A Venn diagram of the alphabets of MetaNetX, eMolecules and ChEMBL datasets (c) Evolution of the cumulative number of new fragments added to the alphabet as a function of the number of molecules processed from the eMolecules database. Each molecule is assigned a value of 0 or 1, depending on whether its atomic signature-ECFP fragments are already present in the alphabet or introduce new elements. (d) Evolution of the percentage of molecules adding new fragments to the alphabet using a sliding window of 1,000 molecules as a function of the number of molecules processed from the eMolecules database.


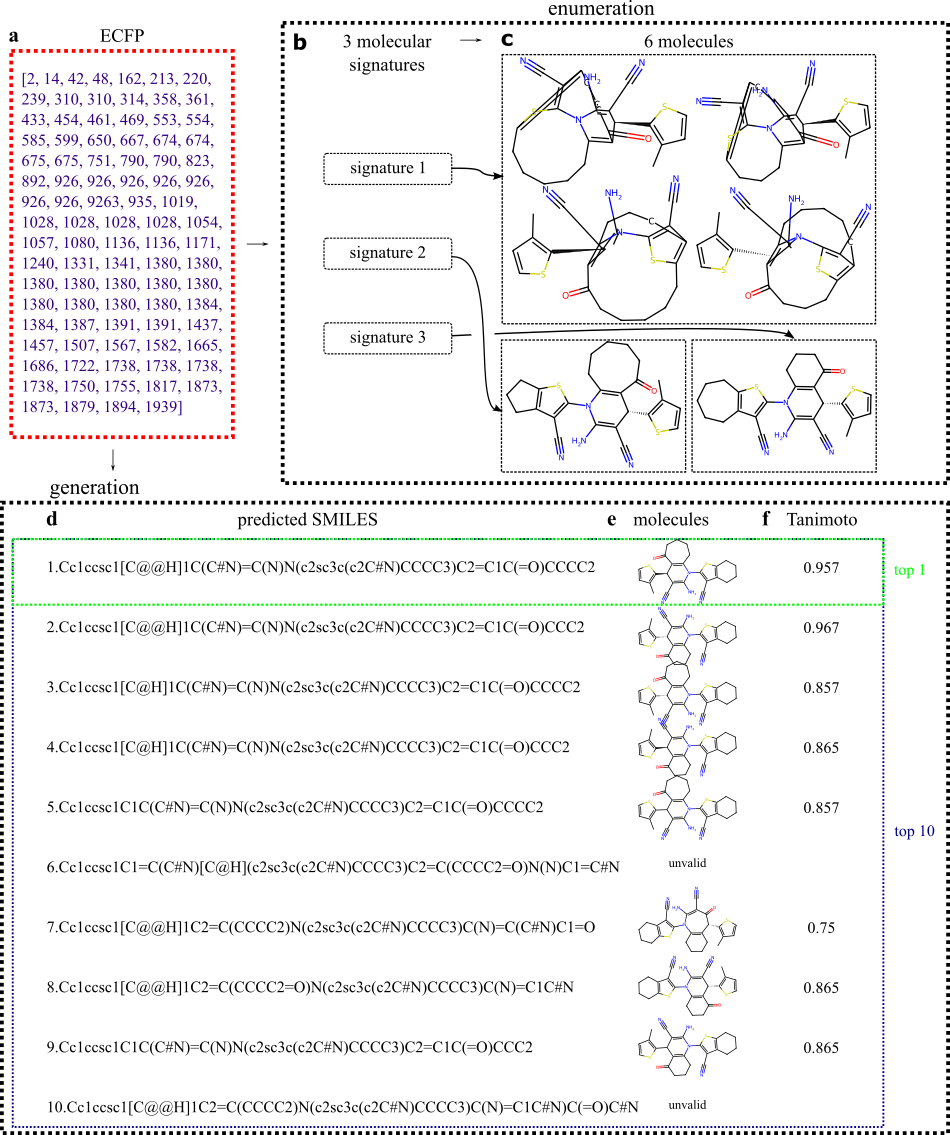


Figure S4. Example of the deterministic enumeration of an ECFP that generates multiple molecular signatures and molecules and where the generative model fails to produce a molecule with the correct ECFP. (a) The ECFP input vector, selected from the eMolecules database, is represented by the counted list of its non-zero components. (b) Using the signature-enumeration algorithm, 3 molecular signatures are obtained. (c) Subsequently, 6 molecules are enumerated using the molecule-enumeration algorithm, each represented alongside their corresponding SMILES strings. (d) The generative model predicts the top 10 SMILES strings based on this input, among which (e) 8 are valid molecules, as verified by RDKit. (f) The Tanimoto coefficients between the predicted molecules and the input ECFP range from 0.957 to 0.75. However, none of the predicted molecules reproduce the exact input ECFP. Specific issues include a valency problem for a carbon atom in the sixth molecule and an excess closing parenthesis in the tenth molecule. Additionally, the fifth and ninth molecules lack stereochemical information.


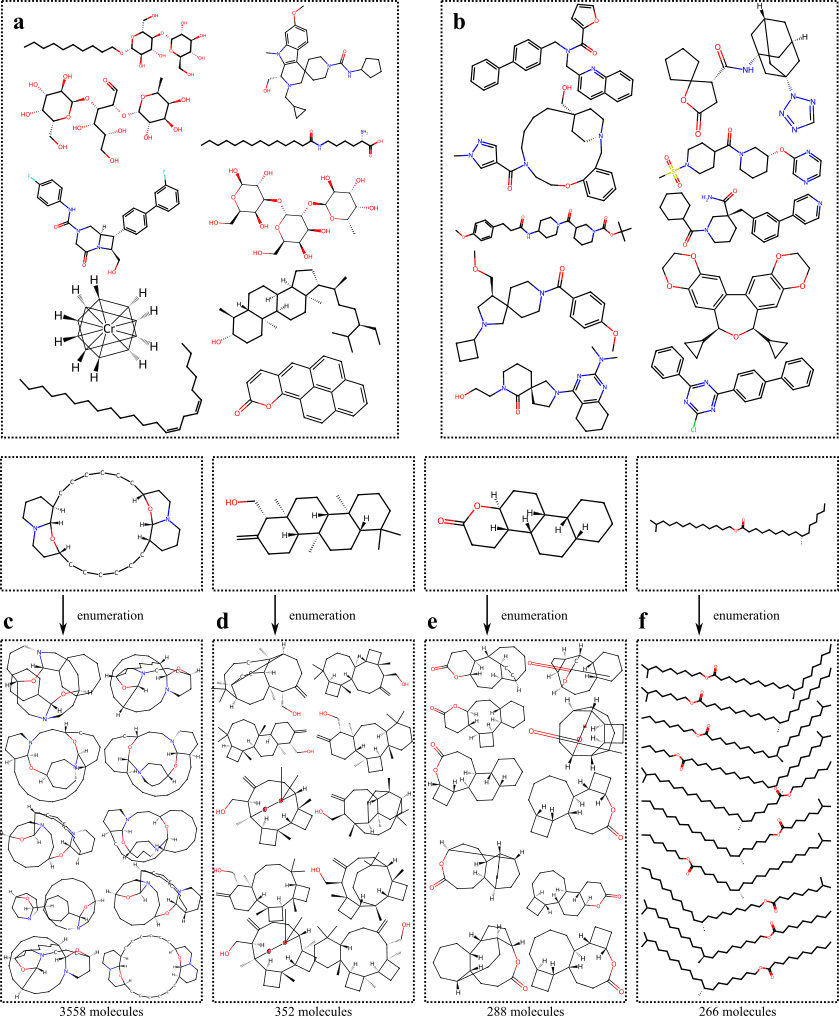


Figure S5. Molecules not reconstructed by the deterministic enumeration algorithm from their ECFPs due to threshold settings imposed to limit computation times and molecules from the MetaNetX database that produce a lot of new molecules through the deterministic enumeration. (a–b) Ten molecules randomly selected from those that could not be reconstructed: 34 from MetaNetX (a) and 45 from eMolecules (b). (c–f) Four MetaNetX molecules that enumerated the largest numbers of structures: 3,558 (c), 352 (d), 288 (e), and 266 (f), respectively. For each molecule, 10 random enumerated molecules are also presented.


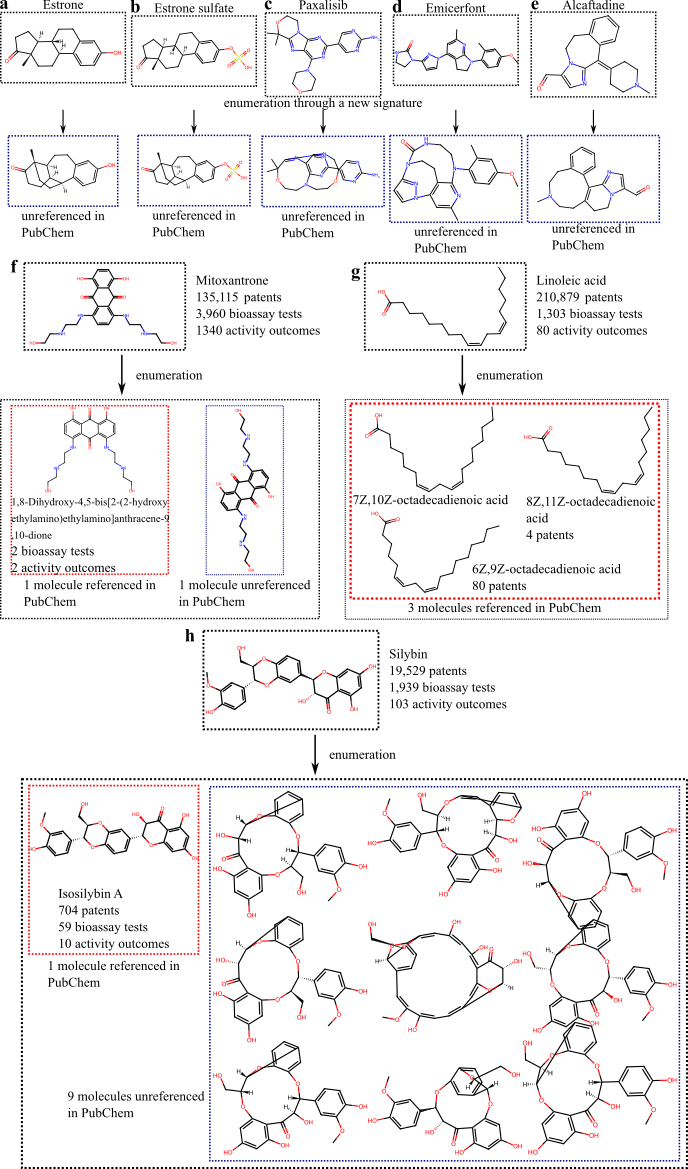


Figure S6. Drug molecules that enumerate new molecules via novel molecular signatures as intermediates and deterministic enumeration algorithm applied on the Mitoxantrone, Linoleic acid and Silybin molecules. The (a) estrone (DB00655), (b) estrone sulfate (DB04574), (c) paxalisib (DB15186), (d) emicerfont (DB12910) and (e) alcaftadine (DB06766) drug molecules that enumerate a new molecule unreferenced in PubChem via a new molecular signature. (f) Mitoxantrone (DB01204) is a U.S. FDA-approved drug for the treatment of secondary progressive, progressive relapsing, and worsening relapsing-remitting multiple sclerosis, yields two enumerated molecules. Among these, one is referenced in PubChem, linked to 2 bioassay tests with 2 activity outcomes, and the other is unreferenced. (g) Linoleic acid (DB14104), a polyunsaturated omega-3 fatty acid commonly found in dietary supplements and approved by the FDA as a drug molecule, yields three enumerated molecules. All three enumerated molecules are referenced in PubChem. Among them, one is associated with 4 patents, while another is linked to 80 patents. (h) Silybin (DB09298), a flavonolignan with hepatoprotective properties, is used to treat toxic liver damage and as an adjunct therapy for chronic conditions such as cirrhosis and hepatitis. It is currently an investigational drug. The enumeration algorithm generates 10 new molecules from Silybin, one of which is referenced in PubChem. This referenced molecule is associated with 704 patents, 59 bioassay tests, and 10 positive activity outcomes. The remaining 9 molecules are not referenced in PubChem.


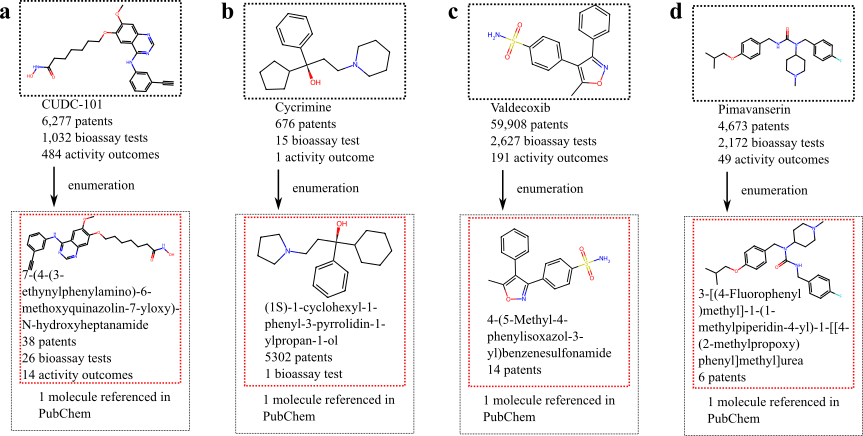


Figure S7. Deterministic enumeration algorithm applied on the CUCDC-101, Cycrimine, Valdecoxib and Pimavanserin molecules. For each of these four drug molecules, the algorithm enumerates one new molecule already referenced in PubChem. (a) CUCDC-101 (DB12174) is currently in Phase I clinical trials for the treatment of various cancers, including liver cancer, breast cancer, gastric cancer, and other tumor types. Its enumerated molecule is associated with 38 patents, 26 bioassay tests, and 14 activity outcomes. (b) Cycrimine (DB00942) is used to restore the balance between acetylcholine and dopamine in the treatment and management of Parkinson's disease. Its enumerated molecule is linked to 5302 patents and one bioassay test. (c) Valdecoxib (DB00580) is a pain-relief medication prescribed for arthritis and to alleviate painful symptoms during the menstrual cycle. Its enumerated molecule is associated with 14 patents. (d) Pimavanserin (DB05316) is a U.S. FDA-approved antipsychotic drug used to treat hallucinations and delusions associated with Parkinson's disease. Its enumerated molecule is linked to six patents.


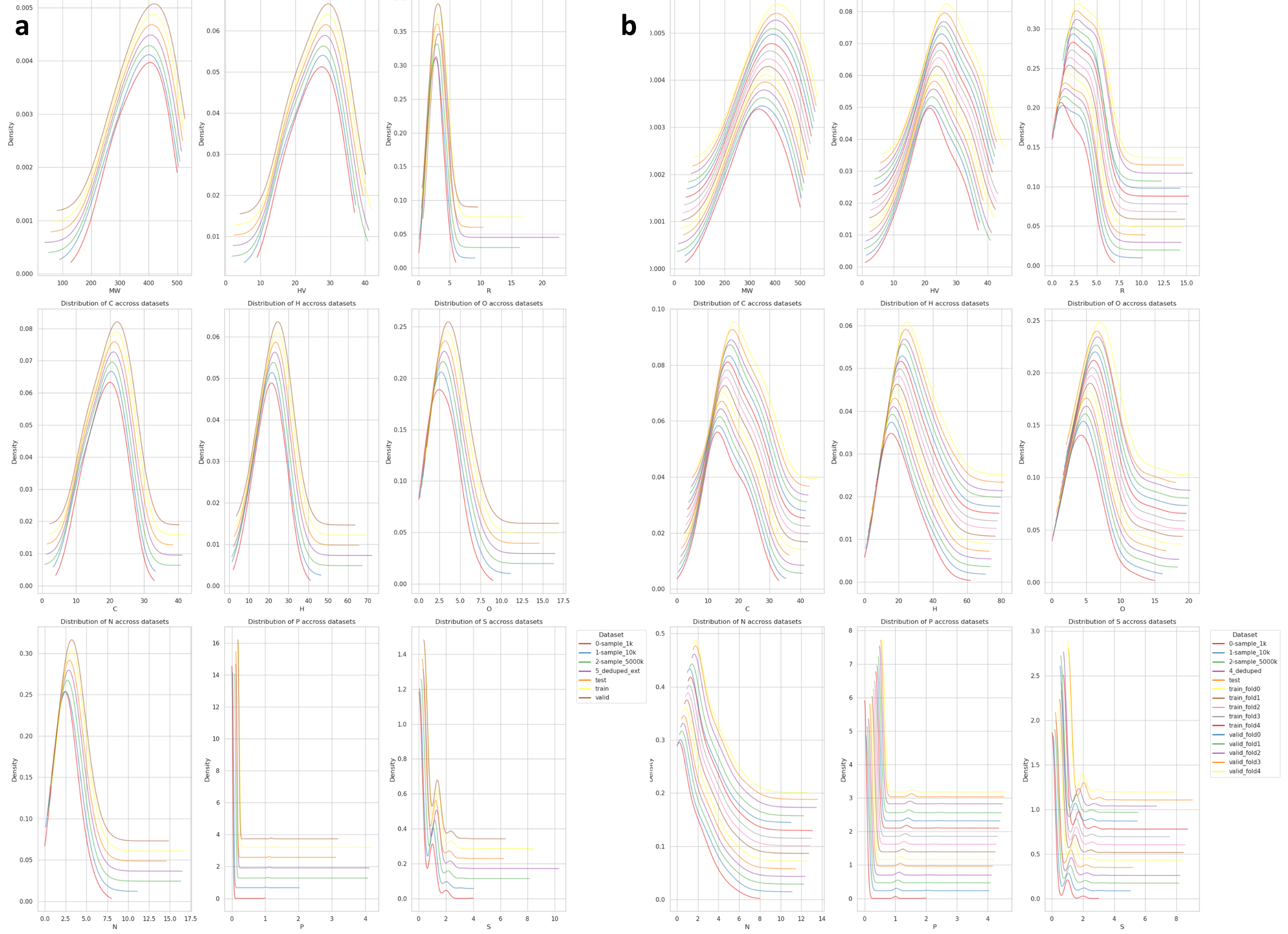


Figure S8. Comparative distributions of molecular properties across eMolecules and MetaNetX subsets. (a) Distributions are shifted for a better readability. For each panel, the x-axis represents the property quantity and the y-axis represents the distribution densities. Properties (from top-left to bottom-right) — molecular weight (MW), number of heavy atom (HV), number of cycles (R), number carbon atoms (C), number of hydrogens (H), number of oxygen atoms (O), number of nitrogens (N), number of phosphate atoms (P), number of sulfur atoms (S). Datasets — “2-sample_5000k”: 5 million molecule subset ; “5_deduped_ext”: complete eMolecules database (17M molecules) ; “test”: test dataset (500k molecules) ; “train” : train dataset (4M molecules) ; “valid”: validation dataset (500k molecules). (b) Distributions are shifted for a better readability. For each panel, the x-axis represents the property quantity and the y-axis represents the distribution densities. Properties (from top-left to bottom-right) — molecular weight (MW), number of heavy atom (HV), number of cycles (R), number carbon atoms (C), number of hydrogens (H), number of oxygen atoms (O), number of nitrogens (N), number of phosphate atoms (P), number of sulfur atoms (S). Datasets — “4_deduped”: complete MetaNetX database (186k molecules) ; “test”: test dataset (18k molecules) ; “train_foldN” : k-fold train datasets (134k molecules each) ; “valid”: k-fold validation datasets (33k molecules each).

Table S1. Pearson correlation between the logarithm of the computation times for the enumeration and generation methods and 30 different molecular complexity measures and descriptors.

|  | **Enumeration** | | | | | | **Generation Top 1** | | **Generation Top 10** | | **Generation Top 100** | |
| --- | --- | --- | --- | --- | --- | --- | --- | --- | --- | --- | --- | --- |
|  | **MetaNetX** | | | **eMolecules** | | | **Meta.** | **eMol.** | **Meta.** | **eMol.** | **Meta.** | **eMol.** |
| **Complexity measure** | CT ecfp_sig | CT sig_mol | CT ecfp_mol | CT ecfp_sig | CT sig_mol | CT ecfp_mol | CT ecfp_mol | CT ecfp_mol | CT ecfp_mol | CT ecfp_mol | CT ecfp_mol | CT ecfp_mol |
| Nb atoms | 0.63 | 0.56 | 0.57 | 0.55 | 0.66 | 0.59 | 0.54 | 0.58 | 0.86 | 0.95 | 0.90 | 0.92 |
| Nb bonds | 0.62 | 0.55 | 0.56 | 0.53 | 0.69 | 0.61 | 0.54 | 0.60 | 0.85 | 0.94 | 0.89 | 0.92 |
| Mol weight | 0.59 | 0.53 | 0.55 | 0.51 | 0.58 | 0.55 | 0.49 | 0.53 | 0.85 | 0.90 | 0.88 | 0.88 |
| ECFP4 sum | 0.63 | 0.56 | 0.59 | 0.57 | 0.68 | 0.60 | 0.55 | 0.60 | 0.83 | 0.93 | 0.87 | 0.91 |
| ECFP4 max | 0.76 | 0.60 | 0.73 | 0.78 | 0.81 | 0.55 | 0.61 | 0.67 | 0.39 | 0.62 | 0.42 | 0.62 |
| ECFP4 prod | 0.52 | 0.43 | 0.68 | 0.60 | 0.52 | 0.45 | 0.67 | 0.61 | 0.69 | 0.80 | 0.73 | 0.78 |
| Sig max | 0.32 | 0.31 | 0.60 | 0.62 | 0.13 | 0.12 | 0.55 | 0.45 | 0.08 | 0.29 | 0.11 | 0.28 |
| Sig prod | 0.26 | 0.22 | 0.66 | 0.59 | 0.26 | 0.22 | 0.71 | 0.57 | 0.25 | 0.47 | 0.28 | 0.46 |
| SMILES length | 0.50 | 0.44 | 0.43 | 0.36 | 0.57 | 0.56 | 0.47 | 0.53 | 0.76 | 0.93 | 0.77 | 0.90 |
| Wiener index | 0.61 | 0.57 | 0.58 | 0.60 | 0.62 | 0.55 | 0.55 | 0.58 | 0.67 | 0.86 | 0.72 | 0.84 |
| Shannon entropy | -0.27 | -0.17 | -0.34 | -0.34 | -0.24 | -0.13 | -0.22 | -0.21 | 0.10 | -0.06 | 0.06 | -0.08 |
| Harary index | 0.60 | 0.54 | 0.54 | 0.51 | 0.68 | 0.61 | 0.53 | 0.59 | 0.84 | 0.94 | 0.88 | 0.92 |
| LogP | 0.22 | 0.23 | 0.40 | 0.38 | 0.39 | 0.29 | 0.37 | 0.34 | 0.10 | 0.43 | 0.14 | 0.42 |
| TPSA | 0.27 | 0.19 | 0.02 | 0.05 | 0.29 | 0.32 | 0.20 | 0.24 | 0.47 | 0.62 | 0.46 | 0.60 |
| H-bond donors | 0.26 | 0.17 | 0.09 | 0.08 | 0.07 | 0.06 | 0.10 | 0.07 | 0.32 | 0.17 | 0.31 | 0.17 |
| H-bond acceptors | 0.30 | 0.24 | 0.07 | 0.09 | 0.36 | 0.37 | 0.18 | 0.27 | 0.52 | 0.63 | 0.51 | 0.61 |
| Rotatable bonds | 0.37 | 0.30 | 0.38 | 0.43 | 0.28 | 0.27 | 0.34 | 0.30 | 0.22 | 0.56 | 0.24 | 0.55 |
| Ring count | 0.29 | 0.28 | 0.31 | 0.23 | 0.68 | 0.54 | 0.40 | 0.51 | 0.49 | 0.62 | 0.52 | 0.61 |
| Fraction Csp3 | 0.19 | 0.20 | 0.21 | 0.21 | -0.18 | -0.03 | -0.14 | -0.07 | 0.07 | -0.15 | 0.06 | -0.14 |
| Balaban index | -0.17 | -0.19 | -0.29 | -0.20 | -0.60 | -0.46 | -0.42 | -0.46 | -0.24 | -0.61 | -0.29 | -0.61 |
| Bertz complexity | 0.44 | 0.38 | 0.35 | 0.32 | 0.70 | 0.58 | 0.48 | 0.53 | 0.73 | 0.87 | 0.77 | 0.85 |
| FCFP4 complexity | 0.33 | 0.31 | 0.10 | 0.12 | 0.53 | 0.52 | 0.20 | 0.32 | 0.73 | 0.79 | 0.74 | 0.76 |
| Bottcher complexity | 0.48 | 0.43 | 0.30 | 0.26 | 0.60 | 0.56 | 0.35 | 0.43 | 0.77 | 0.85 | 0.78 | 0.84 |
| Proudfoot complexity | 0.47 | 0.44 | 0.32 | 0.31 | 0.58 | 0.57 | 0.34 | 0.43 | 0.81 | 0.88 | 0.84 | 0.86 |
| SPS | 0.38 | 0.36 | 0.37 | 0.29 | 0.49 | 0.51 | 0.35 | 0.47 | 0.53 | 0.63 | 0.54 | 0.62 |
| nSPS | 0.16 | 0.17 | 0.18 | 0.09 | 0.09 | 0.14 | 0.01 | 0.11 | 0.28 | 0.00 | 0.28 | 0.01 |
| sascore | 0.08 | 0.10 | -0.02 | -0.04 | -0.07 | 0.07 | -0.16 | -0.02 | 0.22 | -0.01 | 0.21 | -0.01 |
| scscore | 0.42 | 0.39 | 0.25 | 0.26 | 0.49 | 0.48 | 0.17 | 0.31 | 0.65 | 0.59 | 0.68 | 0.58 |
| mc1 | -0.12 | -0.09 | -0.30 | -0.30 | -0.37 | -0.20 | -0.27 | -0.27 | 0.10 | -0.14 | 0.06 | -0.16 |
| mc2 | 0.40 | 0.36 | 0.28 | 0.23 | 0.21 | 0.27 | 0.18 | 0.20 | 0.69 | 0.55 | 0.70 | 0.52 |

Table S2. Drug molecules enumerating 20 or more new molecules with identical ECFP.

| **Drug name** | **DrugBank ID** | **# New enumerated molecules** |
| --- | --- | --- |
| Lupeol | DB12622 | 95 |
| Betulin | DB16890 | 95 |
| Betulinic Acid | DB12480 | 95 |
| (1R,2R,3S,4R,6S)-3,4,6-Trihydroxy-5-{[(S)-hydroxy(3-hydroxy-2-oxopropoxy)phosphoryl]oxy}-1,2-cyclohexanediyl bis[dihydrogen (phosphate)] | DB02028 | 47 |
| 1-O-Octyl-2-Heptylphosphonyl-Sn-Glycero-3-Phosphoethanolamine | DB03565 | 47 |
| Zuranolone | DB15490 | 35 |
| Glucosaminyl-(Alpha-6)-D-Myo-Inositol | DB03779 | 32 |
| Squalane | DB11420 | 32 |
| 2'-(4-Dimethylaminophenyl)-5-(4-Methyl-1-Piperazinyl)-2,5'-Bi-Benzimidazole | DB04011 | 26 |
| Estrane-3,17-dione | DB04693 | 23 |
| 5alpha-androstane-3alpha,17beta-diol | DB01530 | 23 |
| Stanolone | DB02901 | 23 |
| 5-Alpha-Androstane-3-Beta,17beta-Diol | DB03882 | 23 |
| 5beta-dihydrotestosterone | DB07447 | 23 |
| 5alpha-androstane-3beta,17alpha-diol | DB03926 | 23 |
| Allolactose | DB04116 | 23 |
| 3,20-Pregnanedione | DB07557 | 23 |
| Eltanolone | DB12308 | 23 |
| Sepranolone | DB12972 | 23 |
| Brexanolone | DB11859 | 23 |
| Hydroxydione | DB08956 | 23 |
| Ganaxolone | DB05087 | 23 |
| Stanolone acetate | DB13951 | 23 |
| Obeticholic acid | DB05990 | 23 |
| 5-ALPHA-PREGNANE-3-BETA-OL-HEMISUCCINATE | DB08510 | 23 |
| Ginsenosides | DB14152 | 23 |
| Ursolic acid | DB15588 | 23 |

Table S3. Statistics on tokens extracted from eMolecules dataset.

|  | **Unique tokens** | | **Item length (nb. tokens per percentile)** | | |
| --- | --- | --- | --- | --- | --- |
| **Type** | All | Kept | 5 % | 50 % | 95 % |
| **SMILES** | 142 | 47 | 22 | 42 | 58 |
| **ECFP** | 2,052 | 2,052 | 37 | 71 | 97 |

Table S4. Bioassay test references for enumerated molecules of example drug molecules.

| **Input drug molecule** | **Enumerated molecule** | |
| --- | --- | --- |
|  | **PubChemId** | **Bioassay tests** |
| DB15026 | 44221100 | https://pubchem.ncbi.nlm.nih.gov/bioassay/422622  https://pubchem.ncbi.nlm.nih.gov/bioassay/422623  https://pubchem.ncbi.nlm.nih.gov/bioassay/422625  https://pubchem.ncbi.nlm.nih.gov/bioassay/422626  https://pubchem.ncbi.nlm.nih.gov/bioassay/422627  https://pubchem.ncbi.nlm.nih.gov/bioassay/422629  https://pubchem.ncbi.nlm.nih.gov/bioassay/422630 |
|  | 11645581 | https://pubchem.ncbi.nlm.nih.gov/bioassay/422622  https://pubchem.ncbi.nlm.nih.gov/bioassay/422623  https://pubchem.ncbi.nlm.nih.gov/bioassay/422625  https://pubchem.ncbi.nlm.nih.gov/bioassay/422626  https://pubchem.ncbi.nlm.nih.gov/bioassay/422630  https://pubchem.ncbi.nlm.nih.gov/bioassay/422631  https://pubchem.ncbi.nlm.nih.gov/bioassay/482148  https://pubchem.ncbi.nlm.nih.gov/bioassay/1259374  https://pubchem.ncbi.nlm.nih.gov/bioassay/1346556  https://pubchem.ncbi.nlm.nih.gov/bioassay/1508602  https://pubchem.ncbi.nlm.nih.gov/bioassay/1517971  https://pubchem.ncbi.nlm.nih.gov/bioassay/422622 |
| DB01083 | 24971296 | https://pubchem.ncbi.nlm.nih.gov/bioassay/362903  https://pubchem.ncbi.nlm.nih.gov/bioassay/362905  https://pubchem.ncbi.nlm.nih.gov/bioassay/362906  https://pubchem.ncbi.nlm.nih.gov/bioassay/362907  https://pubchem.ncbi.nlm.nih.gov/bioassay/1798446 |
| DB01204 | 44541343 | https://pubchem.ncbi.nlm.nih.gov/bioassay/437514  https://pubchem.ncbi.nlm.nih.gov/bioassay/437515 |
| DB09298 | 11059920 | https://pubchem.ncbi.nlm.nih.gov/bioassay/462333  https://pubchem.ncbi.nlm.nih.gov/bioassay/462334  https://pubchem.ncbi.nlm.nih.gov/bioassay/462335  https://pubchem.ncbi.nlm.nih.gov/bioassay/462336  https://pubchem.ncbi.nlm.nih.gov/bioassay/462338  https://pubchem.ncbi.nlm.nih.gov/bioassay/462339  https://pubchem.ncbi.nlm.nih.gov/bioassay/462340  https://pubchem.ncbi.nlm.nih.gov/bioassay/462341  https://pubchem.ncbi.nlm.nih.gov/bioassay/462342  https://pubchem.ncbi.nlm.nih.gov/bioassay/462344  https://pubchem.ncbi.nlm.nih.gov/bioassay/462345  https://pubchem.ncbi.nlm.nih.gov/bioassay/462346  https://pubchem.ncbi.nlm.nih.gov/bioassay/462347  https://pubchem.ncbi.nlm.nih.gov/bioassay/462348  https://pubchem.ncbi.nlm.nih.gov/bioassay/462350  https://pubchem.ncbi.nlm.nih.gov/bioassay/462353  https://pubchem.ncbi.nlm.nih.gov/bioassay/462354  https://pubchem.ncbi.nlm.nih.gov/bioassay/462355  https://pubchem.ncbi.nlm.nih.gov/bioassay/462356  https://pubchem.ncbi.nlm.nih.gov/bioassay/462357  https://pubchem.ncbi.nlm.nih.gov/bioassay/462358  https://pubchem.ncbi.nlm.nih.gov/bioassay/462359  https://pubchem.ncbi.nlm.nih.gov/bioassay/462360  https://pubchem.ncbi.nlm.nih.gov/bioassay/462361  https://pubchem.ncbi.nlm.nih.gov/bioassay/462362  https://pubchem.ncbi.nlm.nih.gov/bioassay/462363  https://pubchem.ncbi.nlm.nih.gov/bioassay/462367  https://pubchem.ncbi.nlm.nih.gov/bioassay/755105  https://pubchem.ncbi.nlm.nih.gov/bioassay/755106  https://pubchem.ncbi.nlm.nih.gov/bioassay/755108  https://pubchem.ncbi.nlm.nih.gov/bioassay/755112  https://pubchem.ncbi.nlm.nih.gov/bioassay/1129260  https://pubchem.ncbi.nlm.nih.gov/bioassay/1129261  https://pubchem.ncbi.nlm.nih.gov/bioassay/1129263  https://pubchem.ncbi.nlm.nih.gov/bioassay/1129264  https://pubchem.ncbi.nlm.nih.gov/bioassay/1214758  https://pubchem.ncbi.nlm.nih.gov/bioassay/1214760  https://pubchem.ncbi.nlm.nih.gov/bioassay/1239596  https://pubchem.ncbi.nlm.nih.gov/bioassay/1239597  https://pubchem.ncbi.nlm.nih.gov/bioassay/1239598  https://pubchem.ncbi.nlm.nih.gov/bioassay/1409598  https://pubchem.ncbi.nlm.nih.gov/bioassay/1603425  https://pubchem.ncbi.nlm.nih.gov/bioassay/1603426  https://pubchem.ncbi.nlm.nih.gov/bioassay/1603428  https://pubchem.ncbi.nlm.nih.gov/bioassay/1603429  https://pubchem.ncbi.nlm.nih.gov/bioassay/1603430  https://pubchem.ncbi.nlm.nih.gov/bioassay/1603431  https://pubchem.ncbi.nlm.nih.gov/bioassay/1603433  https://pubchem.ncbi.nlm.nih.gov/bioassay/1603434  https://pubchem.ncbi.nlm.nih.gov/bioassay/1603435  https://pubchem.ncbi.nlm.nih.gov/bioassay/1603436  https://pubchem.ncbi.nlm.nih.gov/bioassay/1603437  https://pubchem.ncbi.nlm.nih.gov/bioassay/1603438  https://pubchem.ncbi.nlm.nih.gov/bioassay/1603439  https://pubchem.ncbi.nlm.nih.gov/bioassay/1603440  https://pubchem.ncbi.nlm.nih.gov/bioassay/1603441  https://pubchem.ncbi.nlm.nih.gov/bioassay/1640020  https://pubchem.ncbi.nlm.nih.gov/bioassay/1645758 |
| DB12174 | 24944855 | https://pubchem.ncbi.nlm.nih.gov/bioassay/461250  https://pubchem.ncbi.nlm.nih.gov/bioassay/461251  https://pubchem.ncbi.nlm.nih.gov/bioassay/461252  https://pubchem.ncbi.nlm.nih.gov/bioassay/1341595  https://pubchem.ncbi.nlm.nih.gov/bioassay/1341596  https://pubchem.ncbi.nlm.nih.gov/bioassay/1341597 |
| DB00942 | 207840 | https://pubchem.ncbi.nlm.nih.gov/bioassay/311524 |
